# Efficacy of oncolytic virus in the treatment of intermediate-to-advanced solid tumors: a systematic review and meta-analysis

**DOI:** 10.1128/jvi.00640-25

**Published:** 2025-06-20

**Authors:** Jin-Zhou Xu, Jian-Xuan Sun, Chen-Qian Liu, Ruo-Yan Pan, Na Zeng, Si-Han Zhang, Ye An, Meng-Yao Xu, Xing-Yu Zhong, Si-Yang Ma, Hao-Dong He, Jia Hu, Shao-Gang Wang, Qi-Dong Xia

**Affiliations:** 1 Department and Institute of Urology, Tongji Hospital, Tongji Medical College, Huazhong University of Science and Technology 12403, Wuhan, China; College of Agriculture & Life Sciences, University of Arizona, Tucson, Arizona, USA

**Keywords:** oncolytic virus, intermediate-to-advanced solid tumor, meta-analysis

## Abstract

This meta-analysis assessed oncolytic virus therapy’s efficacy for intermediate-to-advanced solid tumors. We systematically searched PubMed, Cochrane Library, and Embase databases until March 6, 2025. Our review included 87 studies, involving 5,385 individuals, encompassing 75 clinical trials and 12 retrospective studies. Patient response rates were as follows: complete response (CR) 11%, partial response (PR) 18%, stable disease (SD) 32%, and progressive disease (PD) 32%. The overall response rate (ORR) was 29%, and the durable response rate (DRR) was 39%. Comparing oncolytic virus therapy to alternatives, the odds ratio (OR) for overall response was 1.62, and the hazard ratio (HR) for overall survival (OS) was 0.86. This analysis emphasizes the effectiveness of oncolytic virus therapy for intermediate-to-advanced solid tumors, highlighting its promise as a treatment option. These findings offer vital insights into cancer therapy researchers and practitioners.

**IMPORTANCE:**

Although previous meta-analyses on this topic have been published, our research addresses the limitations of previous studies by including missed and newly published studies, updating the results, and conducting extensive subgroup analyses. Our study fills a gap in the literature by providing a comprehensive evaluation of the therapeutic potential of oncolytic viruses for solid tumors.

## INTRODUCTION

Malignant tumors have become the second leading cause of morbidity and mortality worldwide, with increasing incidence and impact each year (1). The primary treatments for malignant tumors, including surgical resection, chemotherapy, and radiotherapy, have shown significant benefits for patients in the early stages of the disease. However, the presence of self-protective mechanisms, such as apoptosis resistance and immune evasion, poses challenges in achieving ideal outcomes for patients with advanced malignant tumors (1). Therefore, there is an imperative to explore new and effective treatment methods for patients with intermediate-to-advanced tumors.

Oncolytic viruses (OVs) are a group of viruses that occur naturally or have been genetically engineered to selectively infect and destroy malignant tumor cells while minimizing side effects on normal cells (2). Common oncolytic virus vectors can be categorized based on their nucleic acid types. These include double-stranded DNA viruses such as adenovirus and herpes simplex virus, double-stranded RNA viruses like reovirus, positive-stranded RNA viruses such as Sacchi virus, and negative-stranded RNA viruses including the measles virus (3). Oncolytic viruses can be classified into two types based on their origin: natural virus strains and genetically modified strains. Natural virus strains have the ability to specifically target tumor cells and selectively replicate within them. Examples of natural virus strains used in oncolytic virotherapy include Newcastle disease virus, myxoma virus, and reovirus (4). The other type is genetically engineered virus strains, which are modified to enhance their ability to target tumor cells. This is achieved by modifying the genes responsible for viral replication in tumor cells and deleting or inactivating key genes involved in viral replication in normal cells. Examples of genetically engineered virus strains used in oncolytic virotherapy include herpes simplex virus, measles virus, adenovirus, and others (4). Currently, there are three OV products approved for market worldwide. RIGVIR, which is a human enterocytopathic orphan virus, is indicated for the treatment of melanoma, colorectal cancer, and other malignancies. Oncorine, an adenovirus-based OV, is used for the treatment of head and neck cancer. T-VEC is another approved OV indicated for the treatment of advanced melanoma. Based on statistics from clinical trials, the viral vectors used in the development of OV drugs worldwide primarily fall into four categories. Adenovirus-based vectors constitute the largest portion with 30 trials (30.9%), followed by herpes simplex virus with 23 trials (23.7%), reovirus with 19 trials (19.6%), and vaccinia virus with 12 trials (12.4%) (5).

With the in-depth study of the anti-tumor mechanism of OVs, people found four main mechanisms. First, OVs can directly cleave tumor cells. Because of the defects in the antiviral response of malignant tumor cells, OVs can specifically infect and replicate in tumor cells, leading to the direct lysis of tumor cells; therefore, through in-depth research on the anti-tumor mechanisms of OVs, scientists have identified four main mechanisms. First, OVs can directly lyse tumor cells. Due to the impaired antiviral response of malignant tumor cells, OVs have the ability to selectively infect and replicate within tumor cells, ultimately resulting in the direct lysis of the tumor cells (6). Second, OVs can induce various forms of immunogenic cell death by triggering the endoplasmic reticulum stress response. This includes necrosis, necrotic apoptosis, immune apoptosis, pyroptosis, and autophagy, which can further stimulate an immune response against the tumor (7). Third, certain OVs, such as herpes simplex virus, vaccinia virus, and vesicular stomatitis virus, have shown the ability to target tumor stromal cells, including tumor-associated fibroblasts, vascular endothelial cells, and pericytes. This targeted infection disrupts the complex tumor structure, leading to tumor necrosis and facilitating the infiltration of immune cells into the tumor microenvironment (TME) (8). Finally, OVs can enhance tumor antigen presentation and immune responses within the TME by inducing antiviral and inflammatory reactions, as well as the production of cytokines (e.g., GM-CSF) and the expression of costimulatory molecules (e.g., TNF-associated activation protein). These immune-stimulating effects result in increased immunogenicity within the TME, breaking immune tolerance and transforming "cold" tumors into “hot” tumors that are more susceptible to immune-mediated destruction (9, 10).

The therapeutic effect of OVs in the treatment of advanced solid tumors can vary. For example, in the treatment of unresectable stage IIIB through IV melanoma, Franke et al. (11) reported a best overall response rate (ORR) of 88.5% with T-VEC, whereas Perez et al. (12) reported the best ORR of 58.6% among their patients. Therefore, in order to comprehensively evaluate the efficacy of OV therapy for solid tumors, we have summarized the findings from published prospective clinical trials and real-world (non-randomized) cohort studies. The purpose of this study was to provide a comprehensive assessment of the current state of OV therapy in the treatment of solid tumors. By employing Response Evaluation Criteria in Solid Tumors (RECIST) and evaluating parameters such as CR, PR, SD, PD, ORR, DRR, OS, and PFS, we aim to shed light on the effectiveness of OVs in this therapeutic approach. This analysis guides us to delve deeper into the topic and explore the outcomes of OV therapy for solid tumors.

## MATERIALS AND METHODS

### Search strategy

Two authors conducted a comprehensive search of relevant articles in PubMed (MEDLINE), Cochrane, and Embase databases up until March 6, 2025. The detailed search strategies can be found in Table S1. The search utilized keywords such as “oncolytic virus,” “oncolytic virotherapy,” and “solid tumor.” To ensure accuracy, a combination of machine and manual deduplication checks were performed, and articles that were not accessible in their original full-text form were excluded. The search was restricted to English-language articles. Additionally, after conducting a thorough analysis and drafting the manuscript, a subsequent literature search was performed to include any newly published studies. Furthermore, we compared the literature included in previous meta-analyses and incorporated any relevant studies that were missing. It is important to note that this meta-analysis has been registered in PROSPERO (CRD42022380652).

### Inclusion criteria

All studies were included based on the PICOS (patients, interventions, comparators, outcomes, and study design) criteria.

- Patients: Individuals diagnosed with intermediate and advanced solid tumors, irrespective of nationality, gender, or race.
- Interventions: Patients who received single-agent therapy or combination therapy with oncolytic viruses (OVs).
- Comparators: Patients who received treatments other than OV therapy.
- Outcomes: The studies should report at least one of the following outcomes: number of complete response (CR), partial response (PR), stable disease (SD), and progressive disease (PD); overall response rate (ORR), durable response rate (DRR), overall survival (OS), and progression-free survival (PFS).
- Study design: Retrospective studies or clinical trials.

### Exclusion criteria

1. Unpublished studies.
2. Duplicated data.
3. Patients who did not receive OV treatment for the first time.

We have included the inclusion/exclusion criteria in Table S2.

### Data extraction and quality assessment

The two authors (Sun and Liu) independently extracted relevant information from the included studies, such as author, year, study type, cancer type, type of OVs, gender, age, outcomes, and more. The quality assessment of the included studies was conducted independently by two authors (Liu and He) using established assessment scales, including the Newcastle–Ottawa Scale (NOS), Jadad scale, and Minors scale. The Newcastle–Ottawa Scale was utilized to assess the quality of retrospective studies. It consists of three major sections (selection, comparability, and assessment of outcome) and nine subsections. A total score of more than 7 indicates good quality. The Jadad scale was employed to evaluate the quality of clinical trials (excluding single-arm clinical trials). It comprises four parts: randomization, allocation concealment, double-blinding, and withdrawals and dropouts. A score higher than 4 is considered indicative of good quality. The Minors scale was used for single-arm clinical trials and includes eight sections. A score higher than 7 is considered indicative of good quality. Detailed scores for each study can be found in Table S3 (Jadad), Table S4 (Minors), and Table S5 (NOS) in the supplementary material.

### Statistical analysis

The meta-analysis was performed using the “meta” package in R v4.0.0 (13). We pooled effect size as odds ratio (OR) and hazard ratio (HR) with 95%CI. The effect sizes were pooled as odds ratios (OR) and hazard ratios (HR) with corresponding 95% confidence intervals (CI). Heterogeneity among the included studies was assessed using the standard Cochrane’s Q test and *I^2^* statistics. A value of *I^2^* >50%, and *P* < 0.1 indicated significant heterogeneity. In cases where substantial heterogeneity was observed, a random-effects model was employed. Conversely, a fixed-effects model was used when heterogeneity was not significant. Sensitivity analysis and cumulative meta-analysis were conducted to explore the potential sources of heterogeneity. Additionally, subgroup analysis was performed based on virus species, country, type of OVs, study type, tumor type, and other relevant factors to identify potential sources of heterogeneity. We also utilized L'Abbé plots and Galbraith plots to illustrate the heterogeneity among the studies. Begg test (14), Egger test (15), trim-and-fill method, and funnel plot were used to estimate the publication bias.

## RESULTS

### Basic information

**Fig 1:**
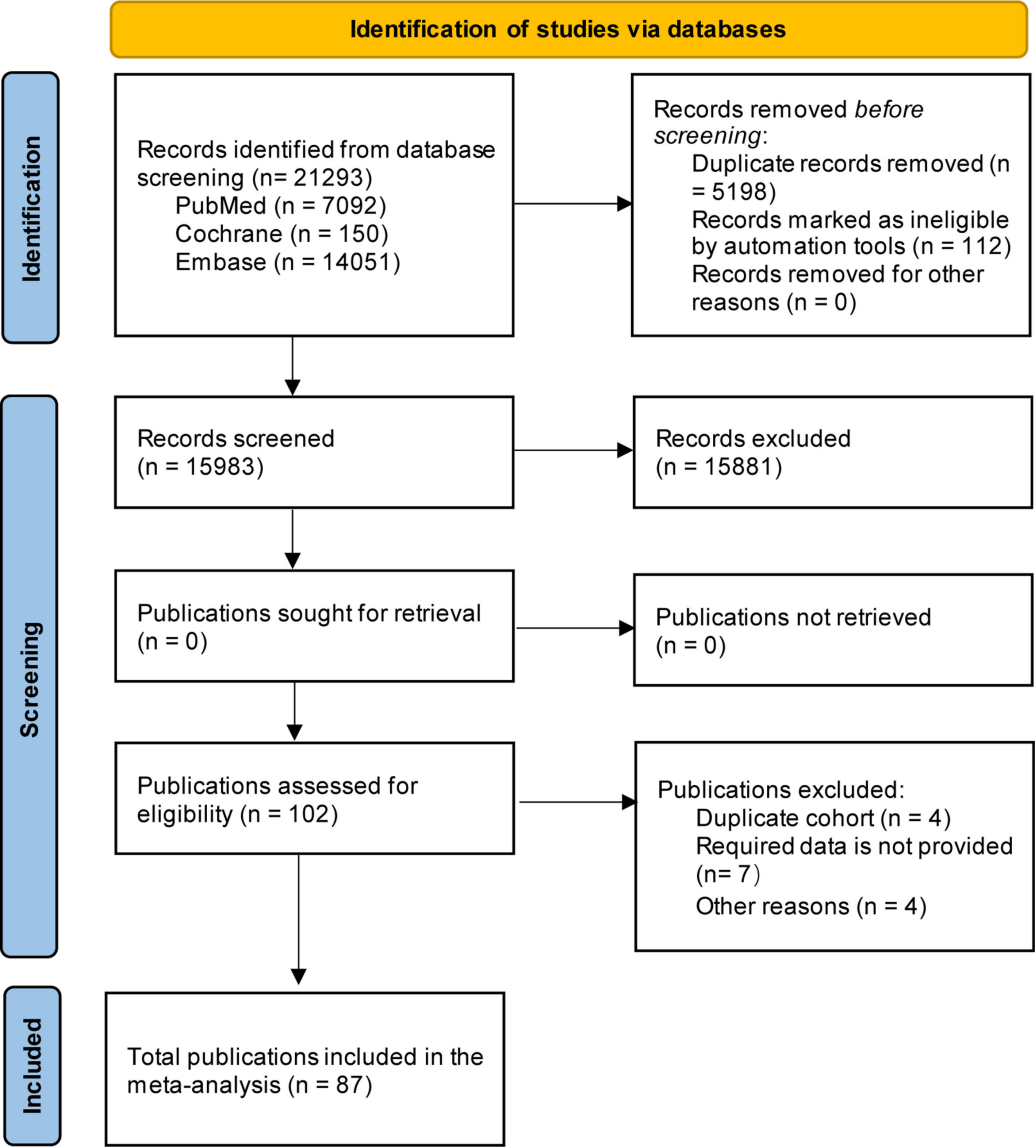
Flow chart illustrating the process of study selection. Inclusion/exclusion criteria can be seen in Table S2.

A total of 21,293 publications were identified through the literature search in PubMed (MEDLINE), Cochrane, and Embase databases up until March 6, 2025. The study selection process was carried out in accordance with the PRISMA flowchart, as shown in Fig. 1. Duplicate articles were removed using automated tools and manual screening. After a thorough review of titles, abstracts, and full-text articles, a total of 87 studies involving 5,385 patients were included in the final analysis (Fig. 1). In particular, the study conducted by Franke et al. (16) was excluded from the analysis because the patient had previously received OV treatment and achieved a CR.

Among the included studies, 73 were clinical trials and 12 were retrospective studies. These studies focused on various types of solid tumors, including 28 studies on melanoma, 9 on hepatocellular carcinoma (HCC), 7 on pancreatic cancer, 4 on lung cancer, 3 on head and neck cancer, and 36 on other tumor types. The basic characteristics of these studies are presented in Table 1 (clinical trials) and Table 2 (retrospective studies). The NOS scores ranged from 7 to 9 for the retrospective studies, indicating good quality. The Jadad scores remained at 4 for the clinical trials, representing good quality as well. The Minor’s scores ranged from 9 to 10, indicating good quality for the included single-arm clinical trials. Furthermore, a total of 32 different OVs were investigated across the included studies.

**TABLE 1:** Characteristics of clinical trials included in this systematic review and meta-analysis^*a*^

Study	Year	Study type	Phase	Randomized	Blind	Country	Cancer type	Tumor characteristics	Age (median or mean)	Male (number, %)	Intervention (treatment vs. control)	Number (treatment vs. control)	Survival	Jadad scores	Minors scores
Khuriet al. (17)	2000	Single arm study			NA	Multi-center	Head and neck cancer	Recurrent	63	86	ONYX-015 vs. none	30 vs. 0		/	11
Tian et al. (18)	2008	Pilot study	II			China	HCC	Unresectable	55	78	rhAd‐p53 + 5-FU + TACE vs. TACE	23 vs. 23	PFS: 9.6 months, OS: 12.8 months	4	/
Nakao et al. (19)	2011	Single arm study	I			Japan	Pancreatic cancer	Unresectable	65	100%	HF10 vs. none	6 vs. 0		/	10
Galanis et al. (20)	2012	Single arm study	II	NA	NA	USA	Melanoma	Metastatic melanoma	65	42.9%	Reolysin vs. none	21 vs. 0	PFS: 45 days (range 13-96 days); OS: 165 days (range 15 days–15.8 months)	/	11
Heo et al. (21)	2013	Non-single arm clinical trial	II	Y	NA	Multi-center	HCC	Advanced	62.9	76.67%	Pexa-Vec (high dose) vs. Pexa-Vec (low dose)	16 vs. 14	OS: 14.1 months vs 6.7 months	4	/
Ye et al. (22)	2014	Non- single arm clinical trial	II	Y	N	Multi-center	Head and neck cancer	Advanced	53	80	E10A + paclitaxel + cisplatin vs. paclitaxel + cisplatin	68 vs. 67	PFS: 7.03 months, OS: 19.1 months	5	/
Villalona-Calero et al. (23)	2016	Single arm study	II		N	USA	Lung cancer	Recurrent or metastatic NSCLC with EGFR activation or KRAS mutations	65	40.54%	Reolysin + paclitaxel + carboplatin vs. nonw	37 vs. 0	The median PFS, OS, and 12-month OS rate were 4 months (95% CI, 2.9-6.1 months), 13.1 months (95% CI, 9.2-21.6 months), and 57% (95% CI, 39%-72%), respectively	/	12
Noonan et al. (24)	2016	Non-single arm clinical trial	II	Y	N	Multi-center	Pancreatic cancer	Recurrent or metastatic pancreatic adenocarcinoma	64	56.16%	Reolysin + paclitaxel and carboplatin vs. paclitaxel and carboplatin	36 vs. 37	Median PFS: 4.9 months (95% CI: 3.0-6.3 months) vs. 5.2 months (95% CI: 2.3-6.2 months); median OS: 7.3 months, 95% CI: 4.8-11.2 months) vs. 8.8 months (95% CI: 6.6-11.8)	5	/
Mahalingam et al. (25)	2016	Single arm study	II			USA	Melanoma	Advanced	56	43	Reolysin vs. none	14 vs. 0	Median PFS: 5.2 months, median OS: 10.9 months	/	12
Xiao et al. (26)	2016	Non-single arm clinical trial	NA	Y	NA	China	Cervical cancer	Advanced	42	0	rhAd‐p53 gene therapy + neoadjuvant chemotherapy vs. cisplatin + vincristine + bleomycin	20 vs. 20		5	/
Puzanov et al. (27)	2016	Single arm study	Ib/II			USA	Melanoma	Unresectable	61	58	T-VEC + Ipilimumab vs. none	19 vs. 0		/	11
Ribas et al. (28)	2017	Single arm study	Ib		N	USA	Melanoma	Surgically unresectable, stage IIIB to IV	58	38.1%	T-VEC + pembrolizumab vs. none	21 vs. 0		/	12
Cohn et al. (29)	2017	Non- single arm clinical trial	IIb	Y	N	USA	Ovarian, tubal, or peritoneal carcinoma	Recurrent or persistent epithelial ovarian, tubal, or peritoneal carcinoma			Reolysin + paclitaxel vs. paclitaxel	52 vs. 48	Median PFS: 4.4 months vs. 4.3 months; median OS: 12.6 months vs. 13.1 months	5	/
Jason Chesney et al. (30)	2017	non- single arm clinical trial	II	Y	N	multi-center	melanoma	unresectable stages IIIB to IV melanoma	65	59.09%	T-VEC + ipilimumab vs. ipilimumab	98 vs. 100	median PFS: 8.2 months (95% CI, 4.2 to 21.5 months vs. 6.4 months (95% CI, 3.2 to 16.5 months)	5	/
Bernhard Josef Eigl et al. (31)	2017	non-single arm clinical trial	II	Y	N	Canada	prostate cancer	advanced	69.1	100%	Reolysin + docetaxel vs. docetaxel	41 vs. 44		5	/
Bernstein et al. (32)	2017	non-single arm clinical trial	II	Y	NA	multi-center	breast cancer	metastatic	61	0	Reolysin + paclitaxel vs. paclitaxel	36 vs. 38	median PFS: 3.78 months, median OS: 17.4 months	6	/
Derek J. Jonker et al. (33)	2017	non- single arm clinical trial	II	Y	NA	Canada	colorectal cancer	metastatic	60	63	Reolysin + FOLFOX6+BEV vs. FOLFOX6 + BEV	51 vs. 52	median PFS: 7 months	5	/
Penelope A. Bradbury et al. (34)	2018	non- single arm clinical trial	II	Y	NA	Canada	lung cancer	advanced or metastatic	63.5	50.66%	Pemetrexed + Reolysin vs. Pemetrexed vs. Docetaxel + Reolysin vs. Docetaxel	38 vs. 37 vs. 39 vs. 38	PFS: 3 months, OS: 7.8 months	5	/
Robert H. I. Andtbacka et al. (35)	2019	non- single arm clinical trial	III	Y	NA	multi-center	melanoma	stage IIIB/C/IV	63	57.34%	T-Vec vs. GM-CSF	295 vs. 141		4	/
Moehler et al. (36)	2019	non-single arm clinical trial	II	Y	N	multi-center	HCC	advanced	60	81.4%	Pexa-Vec plus BSC vs. BSC	86 vs. 43	Median OS: 4.2 vs. 4.4 months; TTP: 1.8 (95% CI: 1.5 to 2.8 months) vs 2.8 months (95% CI: 1.5 months to not unable to evaluate due to censoring)	4	/
Hatem Soliman et al. (37)	2020	single arm study	I			USA	breast cancer	stage T2-3N0-2M0 triple-negative invasive	38	0%	TVEC vs. none	9 vs. 0		/	10
Devalingam Mahalingam et al. (38)	2020	single arm study	Ib	N	N	USA	pancreatic cancer	advanced (unresectable or metastatic) pancreatic ductal adenocarcinoma	64	23.27%	Pelareorep + pembrolizumab vs. none	11 vs. 0	Median PFS: 2.0 months (95% CI:0 to 6.8 months); median OS: 3.1 months (95% CI: 0 to 8.7 months)	/	12
Erin L. Schenk et al. (7)	2020	non- single arm clinical trial	II	Y	Y	multi-center	lung cancer	advanced	63	52	NTX-010 vs. Placebo	26 vs. 24	Median PFS:1.7 months, median OS:13.2 months	7	/
Kevin J. Harrington et al. (39)	2020	single arm study	Ib			multi-center	head and neck cancer	metastatic	62	80.6%	T-VEC + pembrolizumab vs. none	36 vs. 0	PFS: 3.0 months, OS: 5.8 months	/	11
Madhavi Manyam et al. (40)	2021	single arm study	I			USA	ovarian cancer	recurrent or progressive	69	NA	Olvi-Vec vs. none	11 vs. 0	PFS：15.7 week (5.7-34.5）	/	10
Varun Monga et al. (41)	2021	single arm study	IB/II			USA	soft-tissue sarcomas	locally advanced	65	70%	TVEC + standard-of-care neoadju_x0002_vant EBRT + surgery vs. none	30 vs. 0	2 year OS: 57%, CI 37%-72%; 2 year OS: 77%, CI: 57%-88%	/	10
Jacek Hajda et al. (42)	2021	single arm study	II			Germany	pancreatic cancer	stage IV	55.14	57.14%	H-1PV vs. none	7 vs 0	PFS: 72; OS:175; PR: 2	/	10
Yasuhiro Shirakawa et al. (43)	2021	single arm study	I			Japan	oesophageal cancer	stage I-Iva	82	76.92%	OBP-301 + radiotherapy vs. none	13 vs. 0	OS: 16.0 (4,3-NA) months	/	10/
Georgia M Beasley et al. (44)	2021	single arm study	I		N	USA	melanoma	stage IIIB, IIIC, or IV	66	NA	PVSRIPO vs. none	12 vs. 0		/	9/
Victor Moreno et al. (45)	2021	non-single arm clinical trial	I	N	N	multi-center	epithelial ovarian, fallopian tube or primary peritoneal cancer	unresectable, platinum-resistant	63	NA	enadenotucirev plus paclitaxel vs. enadenotucirev	28 vs. 10	PFS: 6.2 (2.8 to 11.1) vs 1.7 (1.2 to 3.5) months	4	/
Tomoki Todo et al. (46)	2022	single arm study	I/II			Japan	glioblastoma	recurrent or progressive	46	61.50%	G47Δ vs. none	13 vs. 0		/	9
Naoya Yamazaki et al. (47)	2022	single arm study	I			Japan	melanoma	advanced	55	27.80%	T- VEC vs. none	18 vs. 0		/	10
Robert H. I. Andtbacka et al. (48)	2022	single arm study	II		N	USA	melanoma	non-resectable stage IIIC or IV	64.7	63.20%	V937(Coxsackievirus A21) intratumoral administration vs. none	57 vs. 0	OS：25.6 (16.7-33.7）months	/	10
ChuanLiang Cui et al. (49)	2022	single arm study	Ib			China	melanoma	unresectable stage IIIC–IV	59	50%	OrienX010 vs. none	26 vs. 0	PFS: 2.9 (1.8-5.7); OS: 19.2 (10.0-27.9)	/	10
Rocio Garcia-Carbonero et al. (50)	2022	single arm study	I			Spain	pancreatic cancer	locally advanced or metastatic, unresectable	66	75	VCN-01 vs. none	16 vs. 0		/	9
Maud Toulmonde et al. (51)	2022	non-single arm clinical trial	II	Y	N	France	soft-tissue sarcoma	advanced	62	40	Pexa-Vec plus metronomic cyclophosphamide vs. metronomic cyclophosphamide	15 vs. 5	PFS: 1.7 months (95% CI 1.1-5.5) vs 7.0 months (95% CI 3.6-9.6); OS: 14.2 months (95% CI 4.1-36.4)	4	/
Lucas Moreno et al. (52)	2023	single arm	I			multi-center	solid tumors	advanced	14	10	T-VEC vs. none	15 vs. 0		/	10
Ann W. Silk et al. (53)	2023	single arm	Ib			multi-center	melanoma	advanced	68.5	27	V937 + pembrolizumab vs none	36 vs. 0	OS:30.9 (20.3-40.5) months, PFS:11.9 (3.4–NR) months	/	11
Jian Guan et al. (54)	2023	single arm	II			USA	non-small cell lung cancer	metastatic	67	16	ADV/HSV-tk + SBRT + pembrolizumab vs. none	27 vs. 0	OS:18.1 months (95% CI, 15.4 to 20.9 months), PFS:7.4 months (95% CI, 5.1 to 9.6 months)	/	10
Vina P. Nguyen et al. (55)	2023	pilot study	I			USA	Breast Cancer	HER2-negative	55	NA	T-VEC vs. none	6 vs. 0		/	10
Katherine E. R. Smith et al. (56)	2023	single arm	I			USA	melanoma	metastatic	66.8	7	VSV-IFNb-TYRP1 vs. none	12 vs. 0	OS:18.9 months	/	9
Sant P. Chawla et al. (57)	2023	single arm	II			USA	sarcoma	advanced	NA	23	T-VEC + Trabectedin + nivolumab vs. none	39 vs. 0	OS:19.3 (95% Confidence Intervals: 12.8 -.), PFS:7.8 (95% Confidence Intervals: 4.1-13.1)	/	10
Robert W. Holloway et al. (58)	2023	single arm	II			USA	ovarian cancer	Platinum-Resistant or Platinum-Refractory	62	NA	Olvi-Vec + platinum-doublet chemotherapy with or without bevacizumab vs. none	27 vs. 0	OS:15.7 (12.3-23.8) months, PFS:11.0 (6.7-13.0) months	/	10
Alexander L. Ling et al. (59)	2023	single arm	I			USA	glioma	High-grade/Recurrent	NA	NA	CAN-3110 vs. none	41 vs. 0	OS:11.6 months (95% CI = 7.8-14.9 months), PFS:1.9 months (95% CI: 1.6-4.5 months)	/	10
Devalingam Mahalingam et al. (60)	2023	single arm	I			USA	pancreatic adenocarcinoma	advanced	63.3	4	pelareorep + pembrolizumab vs. none	13 vs. 0	PFS and OS was 1.9 months and 6.3 months	/	9
Steffan T. Nawrocki et al. (61)	2023	single arm	I			USA	myeloma	relapsed/refractory	NA	9	pelareorep vs. none	14 vs. 0	OS:42.6 months (95% CI, 2.0 to ≥ 69.2 months), PFS:2.6 months (95% CI, 0.9-10.3 months	/	11
Jie Zhang et al. (62)	2023	single arm	NA			China	gastrointestinal malignancy	liver metastasis	59.96	34	OV vs. none	47 vs. 0		/	10
Reinhard Dummer et al. (63)	2023	non-single arm clinical trial	II	Y	N	multi-center	melanoma	advanced	NA	NA	T-VEC then surgery vs. surgery	76 vs. 74	OS: (HR, 0.54; 80% CI, 0.36-0.81), Event-free survival (HR, 0.57; 80% CI, 0.43-0.76)	6	/
Viola Franke et al. (64)	2023	single arm	NA			Netherland	melanoma	IIIB/C-IVM1a unresectable	78.2	9	T-VEC vs. none	10 vs. 0		/	10
Jason A Chesney et al. (65)	2023	non- single arm clinical trial	II	Y	N	multi-center	melanoma	advanced	NA	NA	T-VEC + ipilimumab vs. ipilimumab	98 vs. 100	OS:84.9 vs. 50.1, PFS:13.5 vs. 6.4	5	/
Marwan Fakih et al. (66)	2023	single arm	I			multi-center	epithelial cancer	advanced or metastatic	59	33	enadenotucirev + nivolumab vs. none	51 vs. 0	OS:16, PFS:1.6	/	11
Jeong Heo et al. (67)	2023	single arm	I			multi-center	HCC	refractory advanced	59.3	18	OBP-301 vs. none	20 vs. 0	OS:6.07(26weeks)	/	9
Qiying Zhang et al. (68)	2023	single arm	NA			China	cervical cancer	Persistent, Recurrent, or Metastatic	53	0	H101 vs. none	17 vs. 0	OS:1-year OS rate was 70.6%, PFS:1-year PFS rate was 69.7%	/	10
R. Hecht et al. (69)	2023	single arm	Ib			multi-center	triple-negative breast cancer and colorectal cancer	liver metastases	TNBC: 53 CRC: 62.5	TNBC: 0 CRC: 13	T-VEC + atezolizumab vs. none	36 vs. 0	OS: TNBC: 19.2 months, CRC: 3.8 months, PFS: TNBC: 5.4 months, CRC: 3 months	/	11
Jose Lutzky et al. (70)	2023	single arm	Ib			USA	uveal melanoma	metastatic	65	5	V937 + ipilimumab vs. none	11 vs. 0	PFS:3.7 months	/	10
Santiago Ponce et al. (71)	2023	non- single arm clinical trial	II	Y	N	multi-center	malignant pleural mesothelioma	unresectable	68	9	ONCOS-102 + pemetrexed + cisplatin/carboplatin vs. pemetrexed + cisplatin/carboplatin	15 vs. 14		4	/
Farshad Nassiri et al. (72)	2023	single arm	I/ II			multi-center	glioblastoma	recurrent	53	29	DNX-2401 + pembrolizumab vs.none	49 vs. 0	OS:12.5 months	/	10
Alexander N. Shoushtari et al. (73)	2023	pilot study				multi-center	Melanoma	Anti-PD-1-Resistant Advanced	73	10(	ONCOS-102 + pembrolizumab vs. none	20 vs. 0		/	10
Evanthia Galanis et al. (74)	2024	single arm	I/II			USA	glioma	Grade 3 or 4	53.5	11	MV-CEA vs. none	23 vs. 0	OS:11.6 months (95% CI: 6.4; 17.8)., PFS:3.4 months (95% CI: 2.9, 4.9)	/	9
Aung Naing et al. (75)	2024	single arm	I			multicenter	epithelial cancer	advanced	55	12	NG-350A vs. none	25 vs. 0	OS:8.2 (4.9, 14.4) months, PFS:1.9 (1.7, 4.1) months	/	11
Jiayong Liu et al. (76)	2024	single arm	Ib			China	melanoma	resectable	57	14	T-VEC vs. none	30 vs. 0	mean OS 45.3 months	/	10
Weihai Ning et al. (77)	2024	single arm	I			China	glioma	Non-secreting	55	NA	Ad-TD-nsIL12 vs. none	8 vs. 0	median OS 5.1 months (range 3.1-21.2 months)	/	10
Yi et al. (78)	2024	single arm	NA			China	HCC	refractory or advanced	52.6	16	H101 + nivolumab vs. none	20 vs. 0	OS:15.04 months, PFS:2.69 months	/	11
Maud Toulmonde et al. (79)	2024	single arm	II			France	soft-tissue sarcoma	advanced, ‘cold’	63	6	JX-594 + avelumab + lowdose cyclophosphamide vs. none	15 vs. 0	OS:10.5 months, PFS:1.8 months	/	9
Yandong He et al. (80)	2024	single arm	NA			China	solid tumors	advanced	61.08	4	YSCH-01 vs. none	13 vs. 0	OS:8.62 months, PFS: 4.97 months	/	10
Karie Runcie et al. (81)	2024	single arm	I			USA	pancreatic cancer	advanced	71	6	T-VEC vs. none	9 vs. 0		/	9
Diwakar Davar et al. (82)	2024	single arm	I			multi-center	solid tumors	advanced	63	27	MEDI5395 + durvalumab vs. none	39 vs. 0		/	11
Caroline Robert et al. (83)	2024	single arm	II			multi-center	melanoma	advanced	65	48	T-VEC + pembrolizumab vs. none	72 vs. 0		/	10
Julia Maria Ressler et al. (84)	2025	single arm	II			Austria	cutaneous basal cell carcinomas	difficult-to-resect	74	7	T-VEC + Radiotherapy vs. none	18 vs. 0		/	10
Liping Zhong et al. (85)	2025	single arm	NA			China	solid tumors	refractory	49.8	9	NDV-GT vs. none	20 vs. 0		/	11
Zhichao Tan et al. (86)	2025	single arm	I/II			China	sarcoma	locally advanced or metastatic	NA	9	OH2 with or without HX008 vs. none	26 vs. 0		/	11
Reinhard Dummer et al. (87)	2025	non- single arm clinical trial	I/II	Y		multi-center	melanoma	resectable stage IIIB–D	61	38	pembrolizumab + vibostolimab vs. pembrolizumab + gebasaxturev vs. pembrolizumab	26 vs. 25 vs. 15		4	/
Santeri A Pakola et al. (88)	2025	single arm	I			multi-center	solid tumors	advanced	NA	NA	TILT-123 vs. none	20 vs. 0		/	10
Matthew Stephen Block et al. (89)	2025	single arm	Ia			multi-center	ovarian cancer	resistant or refractory	66	0	TILT-123 + pembrolizumab vs. none	15 vs. 0	OS:6.33(190days), PFS:3.27(98days)	/	10
Xuan Wang et al. (90)	2025	single arm	Ia/Ib			China	melanoma	unresected stage III–IV	59.5	19	OH2 vs. none	29 vs. 0		/	11

^*a*^ NA, not available; vs., versus; BSC, best supportive care; TNBC: triple negative breast cancer; CRC: colorectal cancer; OS, overall survival; PFS, progression free survival; TACE, transhepatic arterial chemoembolization; GM-CSF, 773 granulocyte-macrophage colony-stimulating factor; USA, The United States of America; HCC, hepatocellular carcinoma.

**TABLE 2:** Characteristics of retrospective studies included in this systematic review and meta-analysis^*a*^

Study	Year	Study type	Country	Cancer type	Tumor characteristics	Age (median or mean)	Male (number, %)	Virus species	Intervention (treatment vs. control)	Number (treatment vs. control)	Outcome measures	NOS scores
Jun Dong et al. (91)	2014	retrospective study	China	HCC	stage I-IV	55	136 (91.3)	human adenovirus type 5	rhAd5 vs. no gene therapy	149 vs. 150	RFS：240 (177.9-302.1）days；OS：1526 (1366-1686）days	8
Evalyn E.A.P. Mulder et al. (92)	2022	retrospective study	Netherlands	melanoma	stage III-IV	72	11 (44)	HSV-1	T-VEC vs. none	25 vs. 0		8
Xiao-Jun Lin et al. (93)	2015	retrospective study	China	HCC	unresectable	55	159 (90.86)	human adenovirus type 5	H101 plus TACE vs. TACE	87 vs. 88	OS: HR, 0.593 (0.353-0.995); PFS: HR, 0.461 (0.244-0.870); OS: 12.95 ± 8.36 months vs 12.87 ± 8.28 months; PFS: 10.49 vs 9.72 months	8
Chao-Bin He et al. (94)	2017	retrospective study	China	HCC	intermediate to advanced	54	430 (90.34)	human adenovirus type 5	H101 plus TACE vs. TACE	238 vs. 238	OS: HR, 0.623 (0.492-0.789); 14 months (range 0-65 months) for the TACE group and 17 months (range 2-71 months)	7
Emma H. A. Stahlie et al. (95)	2021	retrospective study	Netherlands	melanoma	stage IIIB-IVM1a	69	40 (43.01)	HSV-1	T-VEC vs. none	93 vs. 0		7
Johannes Kleemann et al. (96)	2021	retrospective study	Germany	melanoma	stage IIIB-IVM1a	83	6 (50)	HSV-1	T-VEC vs. none	12 vs. 0	Median PFS was 20 months (Cl 95% 0-43.8), and median OS was 41 months (Cl 95% NA–NA).	7
Raphael J Louie et al. (97)	2018	retrospective study	multi-center	melanoma	unresectable stage IIIB to IV melanoma	68.8	41 (51.25)	HSV-1	T-VEC vs. none	80 vs. 0		7
Matthew C. Perez et al. (12)	2018	retrospective study	USA	melanoma	unresectable stage IIIB through IV melanoma	75	10 (37.1)	HSV-1	T-VEC vs. none	27 vs. 0		7
Alice Zhou et al. (98)	2019	retrospective study	multi-center	melanoma	metastatic	73	28 (70)	HSV-1	T-VEC vs. none	40 vs. 0	PFS: 10 months	8
Anne Fröhlich et al. (99)	2019	retrospective study	Germany	melanoma	stage IIIB–IV M1c	72.5	6 (43)	HSV-1	T-VEC vs. none	14 vs. 0	PFS: 20 weeks	8
Baocheng Wang et al. (100)	2023	retrospective study	China	lung cancer, breast cancer, gastrointestinal carcinoma, and other	with malignant pleural effusion and ascites	NA	229 (35.6)	H101	H101 vs. none	643 vs. 0		7
Kailan Sierra-Davidson et al. (101)	2025	retrospective study	USA	melanoma	NA	78	37 (53.6)	T-VEC	T-VEC vs. none	69 vs. 0		7

^*a*^ NA, not available; vs., versus; OS, overall survival; PFS, progression free survival; TACE, transhepatic arterial chemoembolization; HCC, hepatocellular carcinoma; HSV-1, herpes simplex virus-1.

### Efficacy of OVs

**Fig 2:**
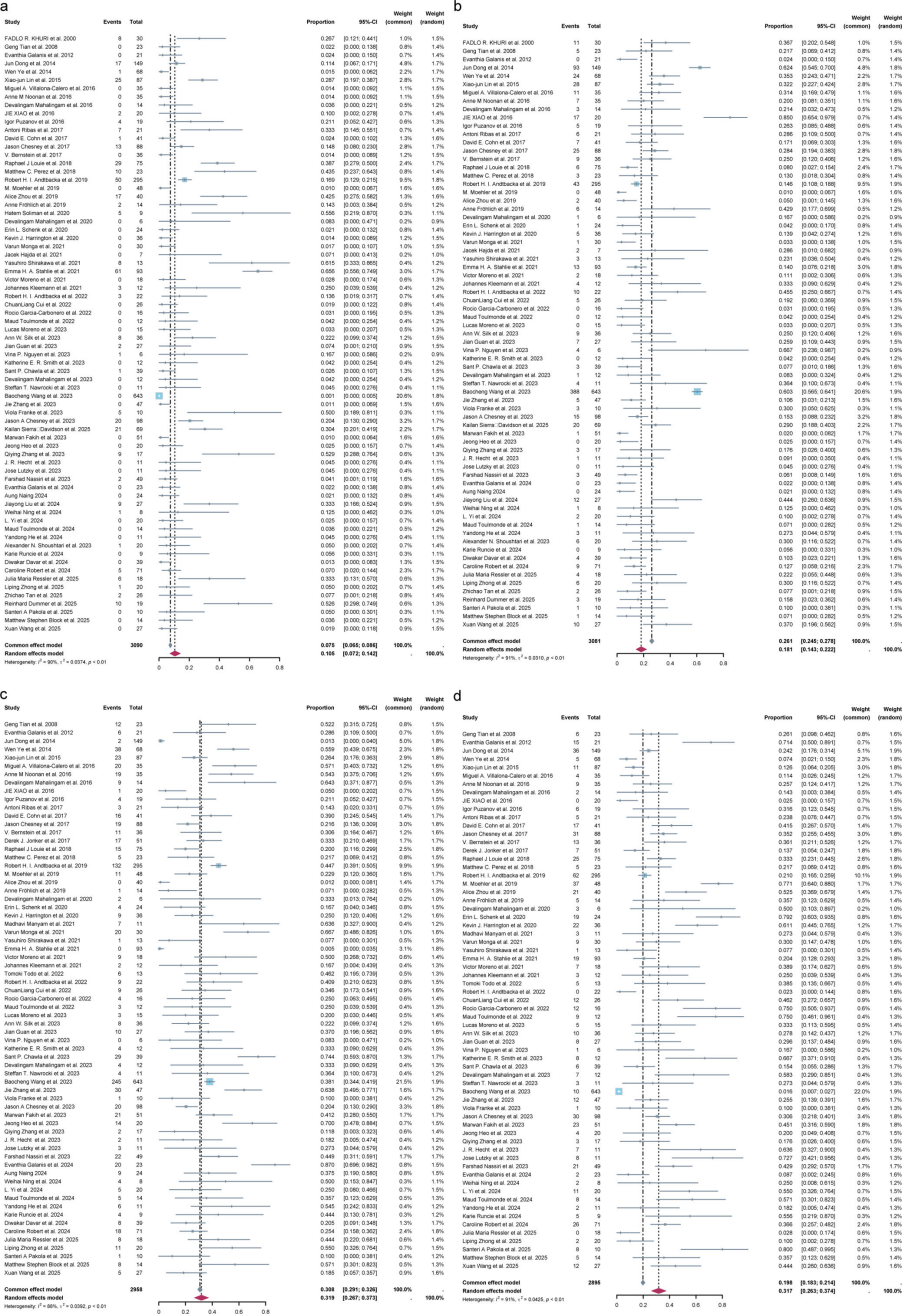
Forest plot demonstrating the CR (a, *n* = 62), PR (b, *n* = 61) SD (c, *n* = 57), and PD (d, *n* = 55) of patients treated with OVs. CR: complete response, PR: partial response, SD: stable disease, PD: progressive disease, OV: oncolytic viruses, CI: confidence intervals.

A total of 66 studies reported the number of patients with CR, PR, SD, or PD. The analysis revealed significant heterogeneity among these studies (*I^2^* = 90%, Fig. 2A; *I^2^* = 91%, Fig. 2B; *I^2^* = 88%, Fig. 2C; *I^2^* = 91%, Fig. 2D; *P* < 0.01), thus the random effects model was employed. Overall, the results showed that 11% of patients achieved CR (95% CI = 7%–14%), 18% achieved PR (95% CI = 14%–22%), 32% achieved SD (95% CI = 27%–37%), and 32% experienced PD (95% CI = 26.3%–37.4%).

**Fig 3:**
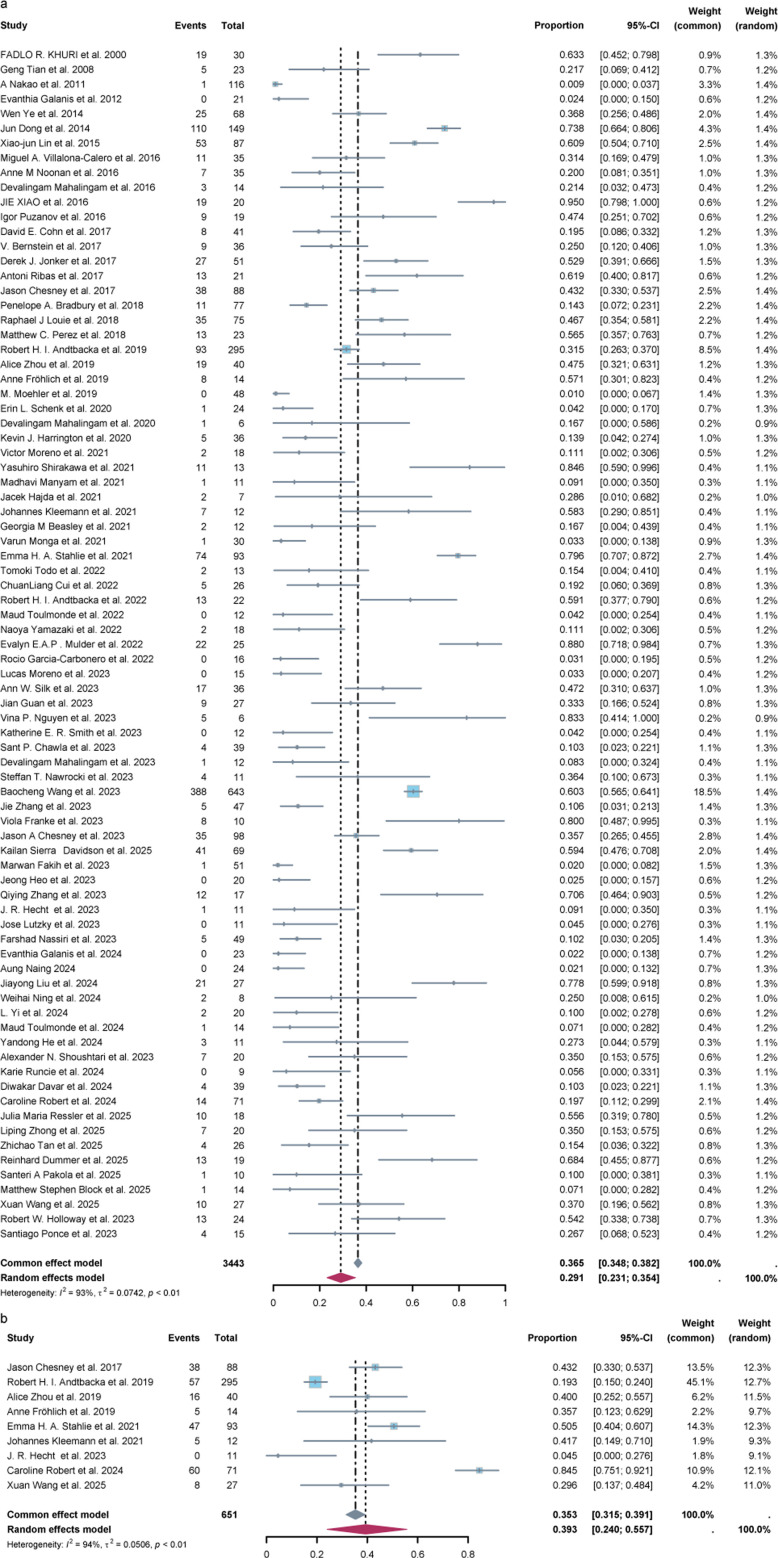
Forest plot presenting the ORR (a, *n* = 81) and DRR (b, *n* = 9) of patients treated with OVs. The ORR of patients was 29% (95% CI = 23%–35%, *P* < 0.01) using the random effects model. The DRR of patients was 39% (95% CI = 24%–56%, *P* < 0.01) using the random effects model. ORR: overall response rate, DRR: durable response rate, CI: confidence intervals.

**Fig 4:**
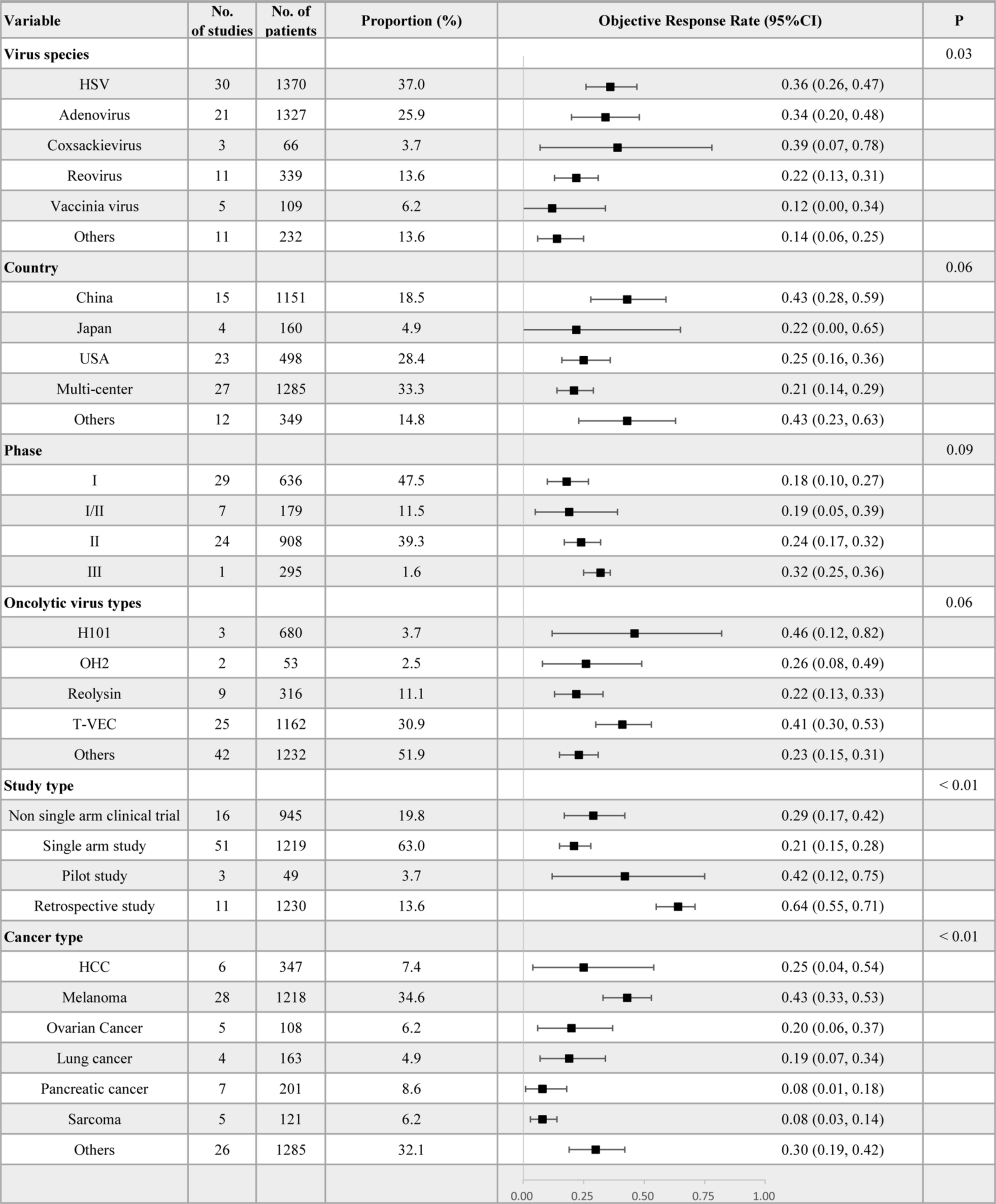
Subgroup analysis of ORR of patients by virus species, country, study phase, OV types, and cancer types. HSV: herpes simplex virus, ORR: overall response rate, OV: oncolytic viruses, CI: confidence intervals, HCC: hepatocellular carcinoma.

**Fig 5:**
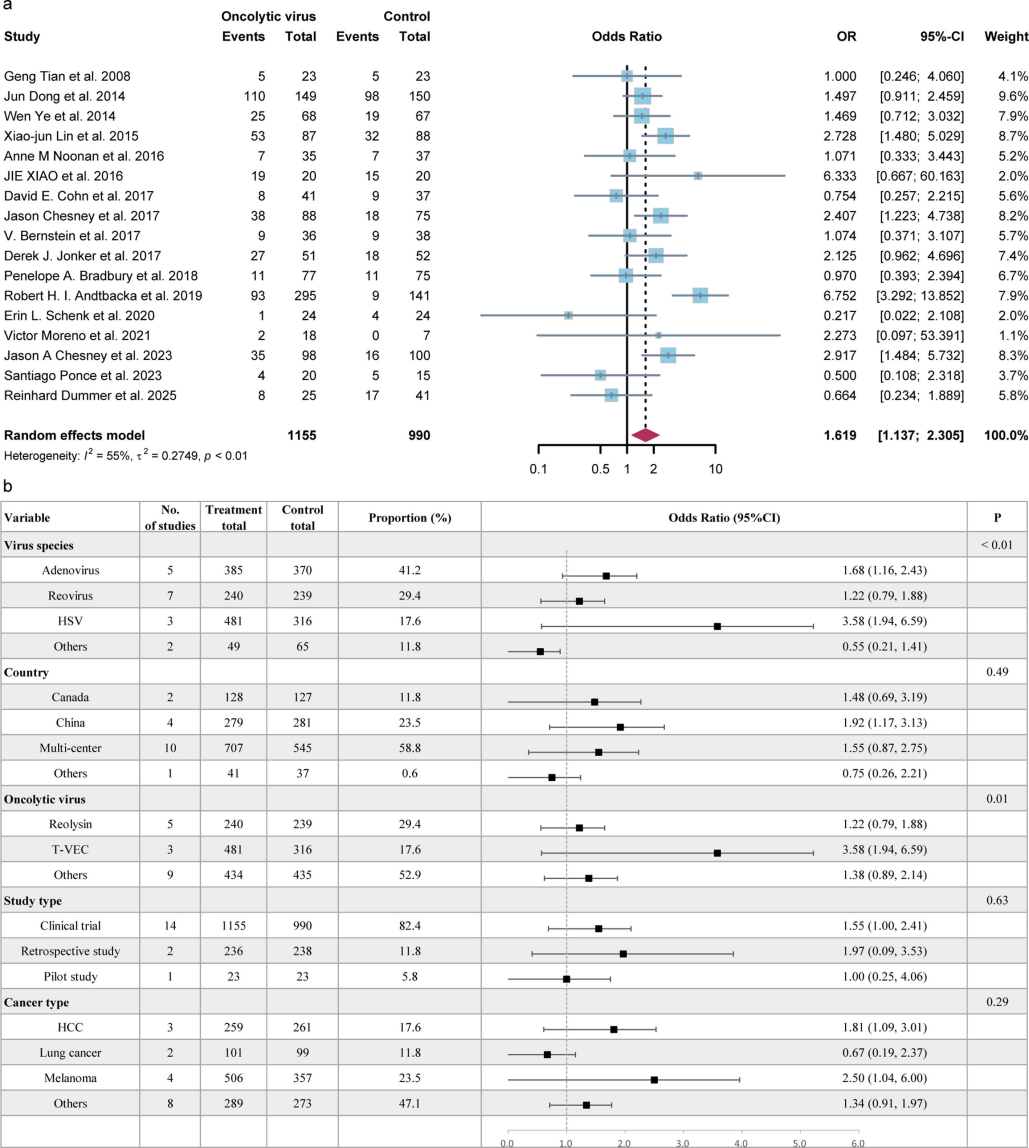
(a)Forest plot of OR of ORR in patients treated with OVs versus no OVs (*n* = 19). The OR of ORR was 1.72 (95%CI = 1.19–2.50, *P* < 0.01) using the random effects model. (b) Subgroup analysis of OR of ORR based on virus species, country, OV types, study types, and cancer types. HSV: herpes simplex virus, OR: odds ratio, ORR: overall response rate, OV: oncolytic viruses, HCC: hepatocellular carcinoma.

We also conducted calculations for the ORR and DRR of patients receiving OV treatment. In the random effects model, the ORR was determined to be 29% (95% CI = 23%–35%), whereas the DRR was found to be 39% (95% CI = 24%–56%), as shown in Fig. 3. In the subgroup analysis of ORR, it was observed that patients treated with herpes simplex virus (HSV) and Coxsackievirus had a higher ORR (36%, 95%CI = 26%–47%; 39%, 95%CI = 7%–78%) compared with other virus species. Additionally, T-VEC and H101 demonstrated a higher ORR (41%, 95%CI = 30%–53%; 46%, 95%CI = 12%–82%) compared with other types of oncolytic viruses (Fig. 4). Moreover, the odds ratio (OR) of ORR comparing OV treatment with other non-OV treatments was calculated and found to be 1.62 (95% CI = 1.14-2.31), indicating a favorable effect of oncolytic virus treatment (Fig. 5A). Significant heterogeneity (*I^2^* = 55%) was observed among the included studies. Subgroup analysis was conducted based on virus species, country, type of OVs, study type, and cancer type to explore potential sources of heterogeneity. In Fig. 5B, the subgroup analysis revealed a surprisingly favorable effect of T-VEC treatment (OR = 3.58, 95% CI = 1.94–6.59). However, it is important to note that only three studies were included in this subgroup, and thus, further research is needed to validate this result. Similarly, the subgroup analysis for melanoma also showed a favorable result (OR = 2.50, 95%CI = 1.04–6.00). Furthermore, we constructed univariate meta-regression models using publication year, age, and proportion of males. However, the results did not reveal any significant associations between publication year (*P* = 0.2385), age (*P* = 0.0811), proportion of males (*P* = 0.3429), and ORR. Similarly, no significant associations were found between publication year (*P* = 0.7920), age (*P* = 0.3587), proportion of males (*P* = 0.4570), and the odds ratio of ORR (Fig. S1a-f and Table S6).

**Fig 6:**
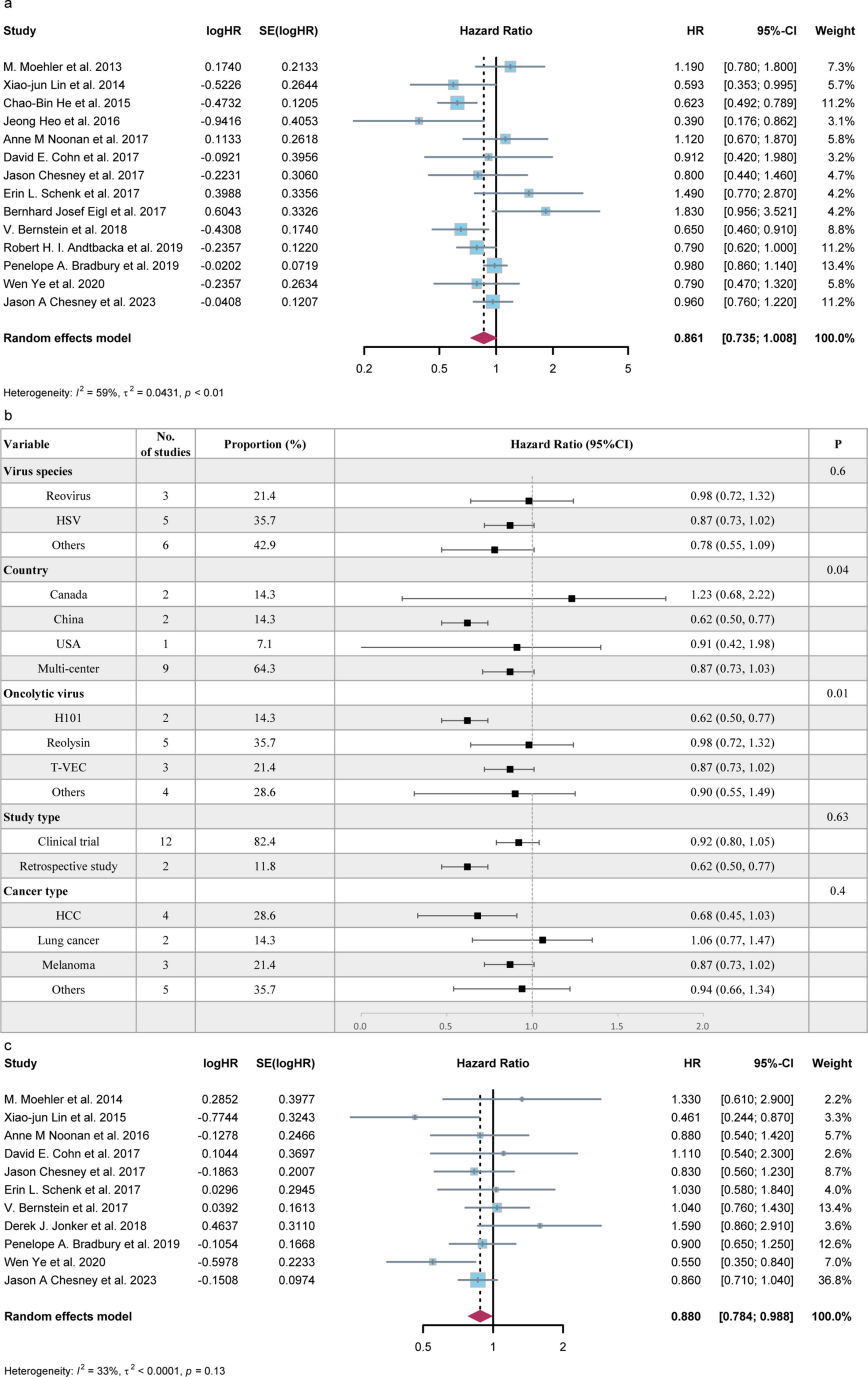
(a) Forest plot of HR of OS in patients treated with OVs versus no OVs (*n* = 14). The HR of OS was 0.85 (95%CI = 0.71–1.02, *P* < 0.01) using random effects model. (b) Subgroup analysis of HR of OS based on virus species, country, OV types, study types, and cancer types. (c) Forest plot of HR of PFS in patients treated with OVs versus no OVs (*n* = 11). The HR of PFS was 0.89 (95%CI = 0.74–1.08, *P* < 0.01) using random effects model. HSV: herpes simplex virus, HR: hazard ratio, OS: overall survival, OV: oncolytic viruses, PFS: progression-free survival, HCC: hepatocellular carcinoma.

A total of 14 studies provided HR for OS, whereas 11 studies provided HR for PFS, comparing patients treated with OVs with those treated with other therapies. The meta-analysis indicated that the treatment of OVs did not have a significant effect on prolonging the overall survival of patients (HR = 0.86, 95%CI = 0.74–1.01, Fig. 6A). Subgroup analysis results are presented in Fig. 6B. However, as shown in Fig. 6C, the effect of OVs on PFS had statistical significance (HR = 0.88, 95%CI = 0.78–0.99). Furthermore, in Fig. S2c and f, a positive relationship was observed between patients' age and OS (*P* = 0.0008) as well as PFS (*P* = 0.0747). This suggests that older individuals have a higher risk of death, and oncolytic viruses may potentially have better therapeutic effects on elderly patients.

### Sensitivity analysis, cumulative meta-analysis, and publication bias

Given the significant heterogeneity observed among the included studies, sensitivity analysis and cumulative meta-analysis were conducted to assess the impact of each study on the overall results. Sensitivity analysis involved omitting or adding each study one by one to determine their influence on the overall outcome. Additionally, funnel plots were constructed to assess publication bias, and the trim-and-fill method, L'Abbé plot, and Galbraith plot were utilized for further evaluation. The results of these analyses, including sensitivity analysis, cumulative meta-analysis, and assessments of publication bias, are presented in the supplemental files for ORR (Fig. S4), DRR (Fig. S4g), odds ratio of ORR (Fig. S5), overall survival (Fig. S6), and progression-free survival (Fig. S7).

After conducting sensitivity analysis and cumulative meta-analysis, all the included studies demonstrated good stability, as shown in Fig. S4a,b, S5a,b, S6a,b and S7a,b. However, the funnel plots for CR, PR, SD, and PD did not exhibit ideal symmetry (Fig. S3), and similar findings were observed for ORR, DRR, odds ratio of ORR, and PFS (Fig. S4d, Fig. 4G, Fig. S5d and S7c). To address potential publication bias, we used the trim-and-fill method to fill in missing studies. For the ORR analysis, 25 missing publications were filled, resulting in a revised ORR of 45% (95% CI = 37%-53%) (Fig. S4c and e), indicating that the missing literature weakened the impact of oncolytic viruses on ORR. In the case of the odds ratio of ORR, three missing publications were filled, yielding a revised odds ratio of 1.90 (95% CI = 1.32–2.73) (Fig. S5c and e), suggesting that the three missing articles also weakened the impact of oncolytic viruses on the odds ratio of ORR. When analyzing PFS, two missing publications were filled, resulting in a revised hazard ratio of 0.83 (95% CI = 0.68–1.02) (Fig. S7d and e), indicating that the missing studies did not significantly affect the overall result. Furthermore, the funnel plot for overall survival (OS) demonstrated good symmetry, with only a few publications lying outside the dashed line (Fig. S6c). No missing articles were filled using the trim-and-fill method (Fig. S6d and e). The Galbraith plot indicated low publication bias (Fig. S4f, S5f,g, S6f, and S7f), and the L'Abbé plot indicated a general agreement among the majority of articles regarding the positive effect of oncolytic viruses in treating intermediate-to-advanced solid tumors (Fig. S5g).

## DISCUSSION

Malignant tumors pose a significant threat to human health and life. Traditional treatments, including surgery, radiotherapy, and chemotherapy, have limitations such as incurability and serious side effects. In recent years, tumor immunotherapy has witnessed significant advancements. By reactivating and sustaining the tumor-immune cycle, it activates the body’s natural anti-tumor immune response, aiming to control and eliminate tumors (102). Currently, anti-tumor immunotherapies encompass various approaches, including immune checkpoint inhibitors, therapeutic antibodies, cancer vaccines, and cell-based therapies (102). Among these approaches, OV therapy holds a prominent position within tumor immunotherapy and has found extensive application in the field of cancer treatment. OV therapy primarily targets solid tumors, such as melanoma, hepatocellular carcinoma, colon cancer, breast cancer, glioma, multiple myeloma, head and neck cancer, and malignant pleural mesothelioma (5). In 2005, China made a significant milestone in the field of oncolytic virus therapy by approving the use of recombinant human adenovirus 5 (H101) in combination with chemotherapy for patients with advanced nasopharyngeal carcinoma. This approval marked the first clinical application of an oncolytic virus product worldwide (103). Andtbacka et al. (48) conducted a phase I clinical trial evaluating the use of Coxsackie virus V937 in the treatment of advanced melanoma. The study reported promising results, with a DRR of 21.1%. Additionally, the 12-month PFS and OS rates reached 32.9% and 75.4%, respectively. These findings suggest the potential effectiveness of Coxsackie virus V937 in improving outcomes for patients with advanced melanoma. Similarly, in a phase I clinical trial evaluating the use of OV monotherapy for patients with unresectable melanoma, the ORR reached 33%. These results demonstrate the significant efficacy of OV treatment as a standalone therapy. However, a phase IIb clinical trial conducted in 2019 evaluating the use of JX-594 showed no significant differences in median OS, total RR, and time to progression (TTP) between the experimental group receiving Pexa-Vec in combination with best supportive care and the control group receiving only best supportive care (36). Therefore, conducting a meta-analysis is crucial to summarize and obtain meaningful results from relevant clinical studies at a statistical level. Upon literature search, it was found that only one meta-analysis on the use of oncolytic virus therapy for intermediate and advanced solid tumors had been published in 2021 (104). After reviewing the existing literature, it became evident that the previous meta-analysis focused more on statistical analysis of research and virus design but did not further summarize and analyze the survival information of patients. As this is a highly active and emerging field, there has been a large number of new clinical studies published recently, and many clinical studies were not included in the previous meta-analysis (5, 105). Therefore, it is necessary to include these missed and newly published studies to provide an updated analysis of the results.

In this meta-analysis, a total of 87 studies were included to explore the effectiveness of OVs in treating patients with intermediate-to-advanced solid tumors. We found that 11% of patients achieved CR (95%CI = 7%–14%), 18% achieved PR (95%CI = 14%–22%), 32% achieved SD (95%CI = 27%–37%), and 32% experienced PD (95%CI = 26.3%–37.4%) after OV treatment. The ORR of patients was 29% (95% CI = 23%–35%), and the DRR was 39% (95% CI = 24%–56%). The OR of ORR was 1.62 (95% CI = 1.14–2.31). Besides, the HR of OS for patients was 0.86 (95% CI = 0.74–1.01), and the PFS was HR = 0.88 (95% CI = 0.78–0.99). However, in the subgroup analysis, it was found that the ORR after T-VEC monotherapy and combined therapy reached 41% (95% CI = 30%–53%), indicating promising therapeutic potential. The higher ORR observed in T-VEC monotherapy and combined therapy could be attributed to the fact that T-VEC is one of the first oncolytic viruses approved by the FDA for treatment (106). It is worth noting that the observed heterogeneity in efficacy across different viruses and cancer types may partly stem from differences in sample size among cancer types. Additionally, the fact that T-VEC has been approved by the FDA may lead to greater confidence in its efficacy in clinical trials,, which may contribute to the observed therapeutic efficacy. T-VEC, belonging to herpes simplex virus type 1 (HSV-1), has been found to induce the release of high mobility group box 1 (HMGB1) protein and calmodulin in human melanoma cells. In addition, it has been shown to promote the proliferation of melanoma tumor antigen in mouse models with melanoma. The injection of T-VEC can selectively induce immunogenic cell death (ICD) of tumor cells, leading to the release of tumor-associated antigens upon tumor cell lysis and triggering a T-cell-mediated immune response (107). Furthermore, in the subgroup analysis, we observed that the ORR among melanoma patients was notably high, reaching 43% (95% CI = 33%–53%). This finding may be attributed to the high immunogenicity of melanoma and its increased sensitivity to immunotherapy, rendering it more responsive to treatment compared to other types of solid tumors (108). It is worth noting that Erin et al. reported that the OS and PFS of patients treated with NTX-010 in SCLC did not seem to be better than those of the placebo group, although the difference was not statistically significant. They also found that viremia was associated with poor prognosis, suggesting that treatment failure might be due to viral clearance (7).

Although OVs have shown clinical benefits in various types and stages of solid tumors, there are still challenges when using OVs as a standalone therapy. The use of OV monotherapy can mitigate issues related to biosafety, complexity, and costs since only one drug needs to be produced and administered. However, to achieve optimal therapeutic effects, OVs must be present in the body for a sufficient duration. Unfortunately, the host immune system can clear the virus from circulation, limiting its presence to a short period. As a result, OV monotherapy may only yield low-to-moderate efficacy (109). To overcome the limitations of single-drug OV treatment, researchers have explored combination therapies, such as radiotherapy, chemotherapy, immune checkpoint inhibitors, CAR-T cell treatments, etc. (106). In a phase II trial targeting recurrent head and neck cancer, a combination therapy involving a modified oncolytic adenovirus ONYX-015, cisplatin, and 5-fluorouracil (5-FU) demonstrated an enhanced anti-tumor effect. The effective rate of the combined therapy was 63% in patients receiving the combination (OVs + cisplatin + 5FU), whereas the effective rate was only 15% in patients receiving ONYX-015 alone. This highlights the potential of combining OVs with traditional chemotherapy agents to achieve improved treatment outcomes (110). CAR-T cell therapy utilizes genetically modified T cells to express CARs, enabling them to recognize and eliminate tumor cells with specific antigens. When combined with gene-modified OVs, CAR-T cell therapy exhibits enhanced efficacy by promoting CAR-T cell infiltration into the TME and improving their anti-tumor activity against solid tumors. This combination approach holds great promise for improving the therapeutic outcomes of CAR-T cell therapy in the treatment of solid tumors (111). A preclinical study combining oncolytic adenovirus Ad-mTNFα-mIL2 (expressing TNF-α and IL-2) with CAR-T cells targeting mesothelin showed significant tumor attenuation in pancreatic ductal adenocarcinoma models (112).

This meta-analysis has several limitations. First, heterogeneity exists due to variations in treatment methods between the experimental and control groups, which could have been addressed through network meta-analysis. Second, the objective response rate could not be calculated due to incomplete data in some articles, limiting the synthesis of results. Third, the safety evaluation of oncolytic viruses was not performed, which is crucial for patients and clinicians. Future studies will include the analysis of side effects. Fourth, some studies provided survival curves without HR values for OS and PFS, leading to the exclusion of survival information. This decision was made to avoid errors associated with the artificial extraction of HR values from survival curves, as outlined by Tierney (113). However, it is important to note that this exclusion may have influenced the results of our analysis. Finally, access to full-text articles may have been restricted, limiting their inclusion and analysis. Although these limitations bring some uncertainties, we believe that their impact on the main conclusions is limited. In future studies, methods such as network meta-analysis and the inclusion of more outcome measures can be adopted to make the research more comprehensive and meticulous.

### Conclusion

In conclusion, this meta-analysis reveals that OVs demonstrate a significant therapeutic effect in the treatment of solid tumors. Specifically, T-VEC has shown to be the most effective type of OV currently. However, no significant association was found between OVs and OS or PFS in patients. Future studies should focus on investigating the side effects of OVs. Furthermore, we encourage more research centers to participate in clinical trials of OVs to generate additional evidence regarding their therapeutic efficacy.

## Data Availability

All the original data were collected from online databases.

## SUPPLEMENTAL MATERIAL

Supplemental figures jvi.00640-25-s0001.docx Figures S1 to S7.

Table S1 jvi.00640-25-s0002.docx Details of search strategy.

Table S2 jvi.00640-25-s0003.docx Inclusion/exclusion criteria.

Table S3 jvi.00640-25-s0004.docx JADAD scale for assessing the quality of clinical trials.

Table S4 jvi.00640-25-s0005.docx Minors scale for assessing the quality of single-arm clinical trials.

Table S5 jvi.00640-25-s0006.doc Newcastle-Ottawa scale (NOS) for assessing the quality of cohort studies.

Table S6 jvi.00640-25-s0007.docx Meta-regression analysis of factors affecting heterogeneity for the association between OVs use and cancer risk.

ASM does not own the copyrights to Supplemental Material that may be linked to, or accessed through, an article. The authors have granted ASM a non-exclusive, world-wide license to publish the Supplemental Material files. Please contact the corresponding author directly for reuse.
