# Supplementary material for: Efficacy of oncolytic virus in the treatment of intermediate-to-advanced solid tumors: a systematic review and meta-analysis
Source: J Virol. 2025 Jun 20;99(7):e00640-25. doi: 10.1128/jvi.00640-25 (PMC12282134; doi:10.1128/jvi.00640-25)
Supplement: Supplemental figures — Figures S1 to S7. [file jvi.00640-25-s0001.docx]

**Supplement Figure Legend**


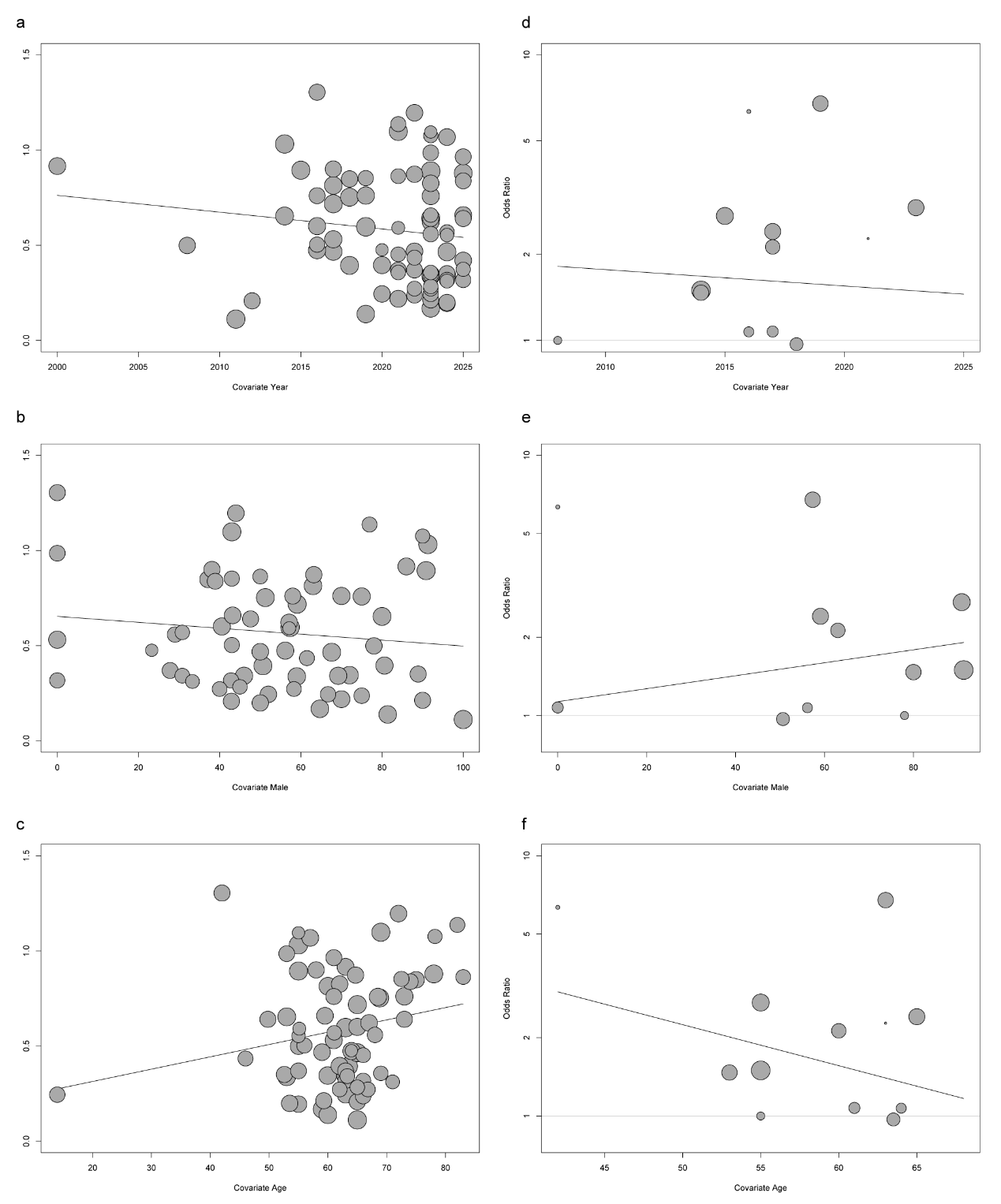


Figure S1: Univariate meta-regression model using (a-b) publication year, (c-d) age, and (e-f) the proportion of male.


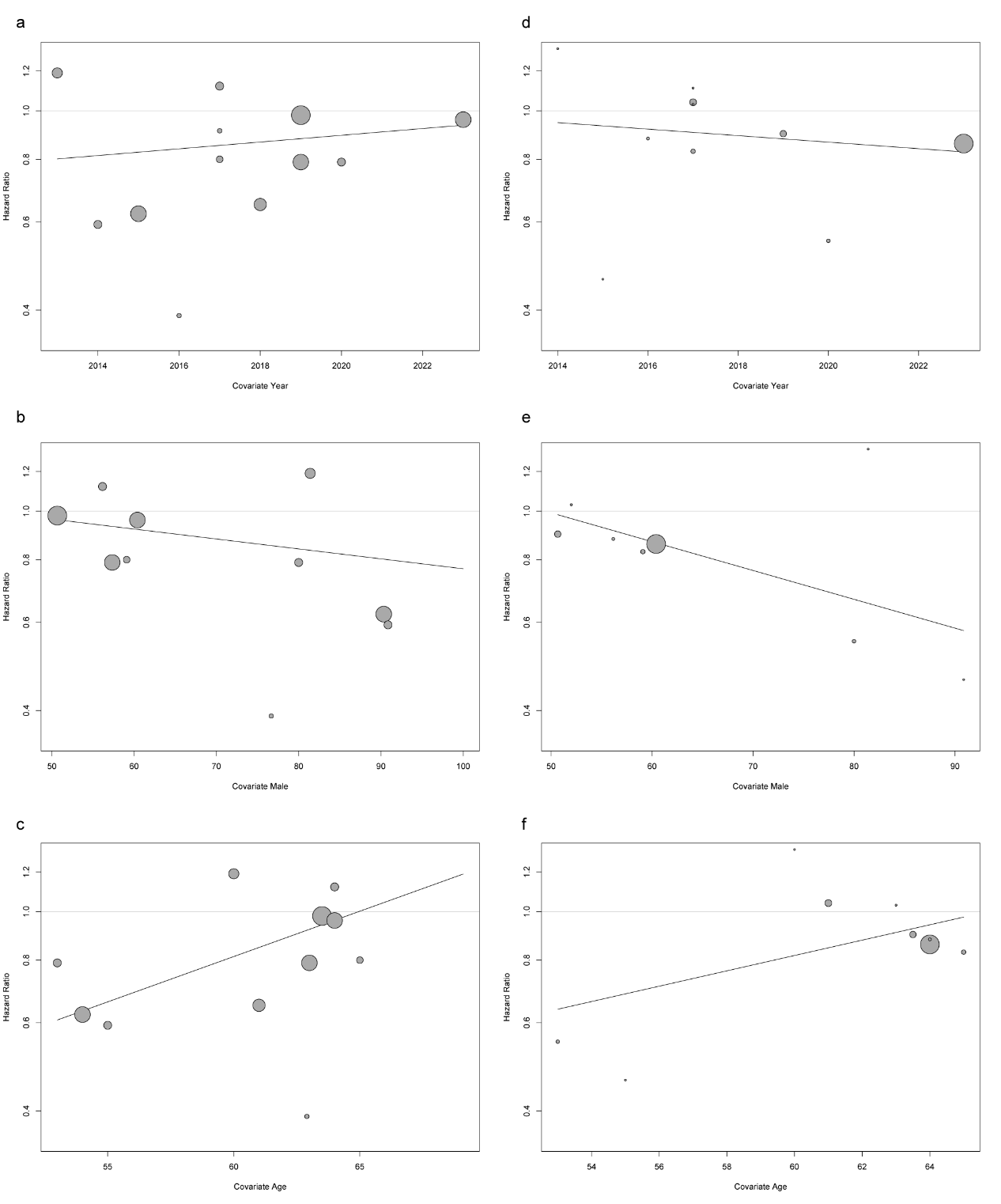


Figure S2: Hazard ratio of OS and PFS in relation to (a, d) publication year, (b, e) the proportion of male, and (c, f) age.


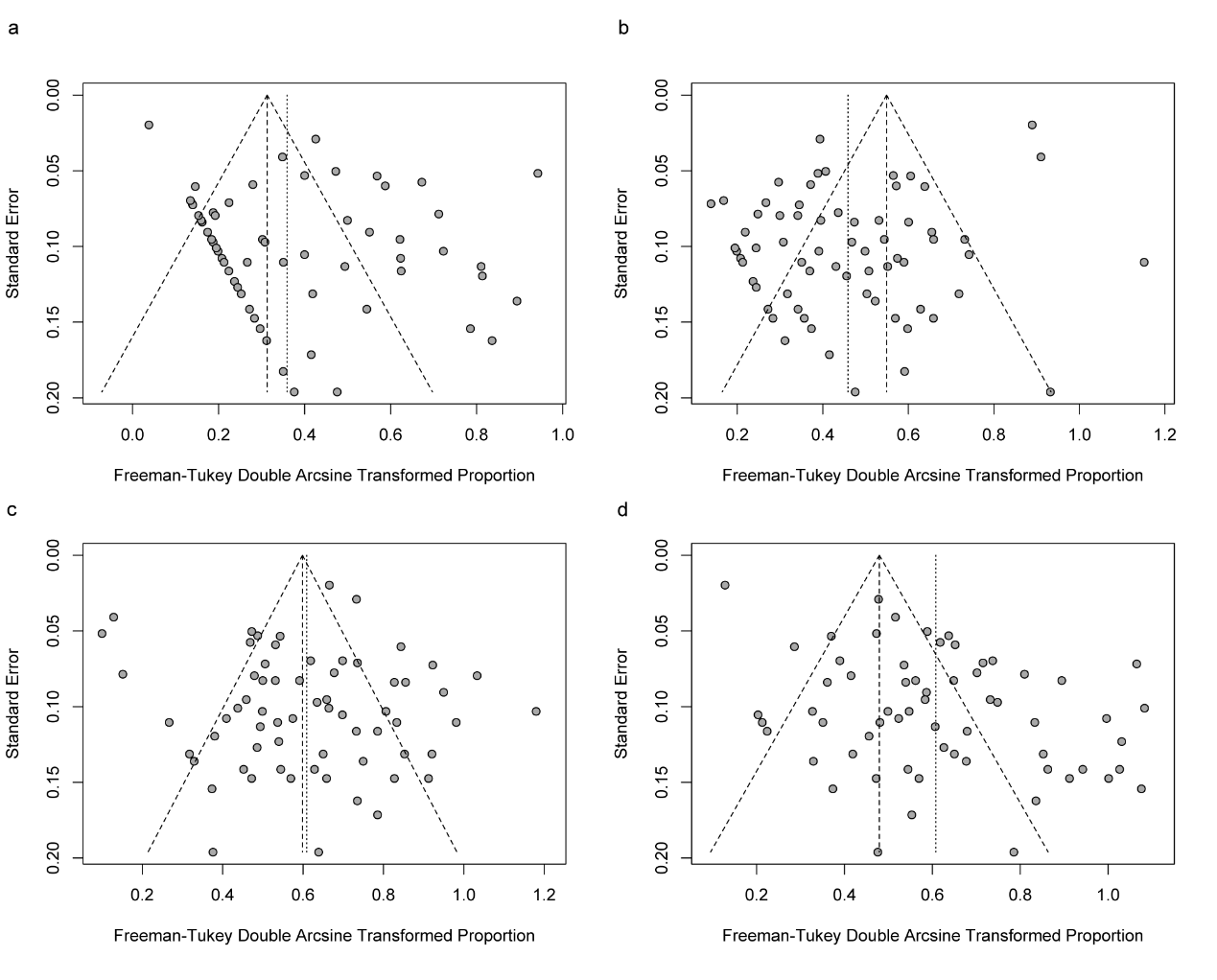


Figure S3: Funnel plot of (a) CR, (b) PR, (c) SD, and (d) PD.


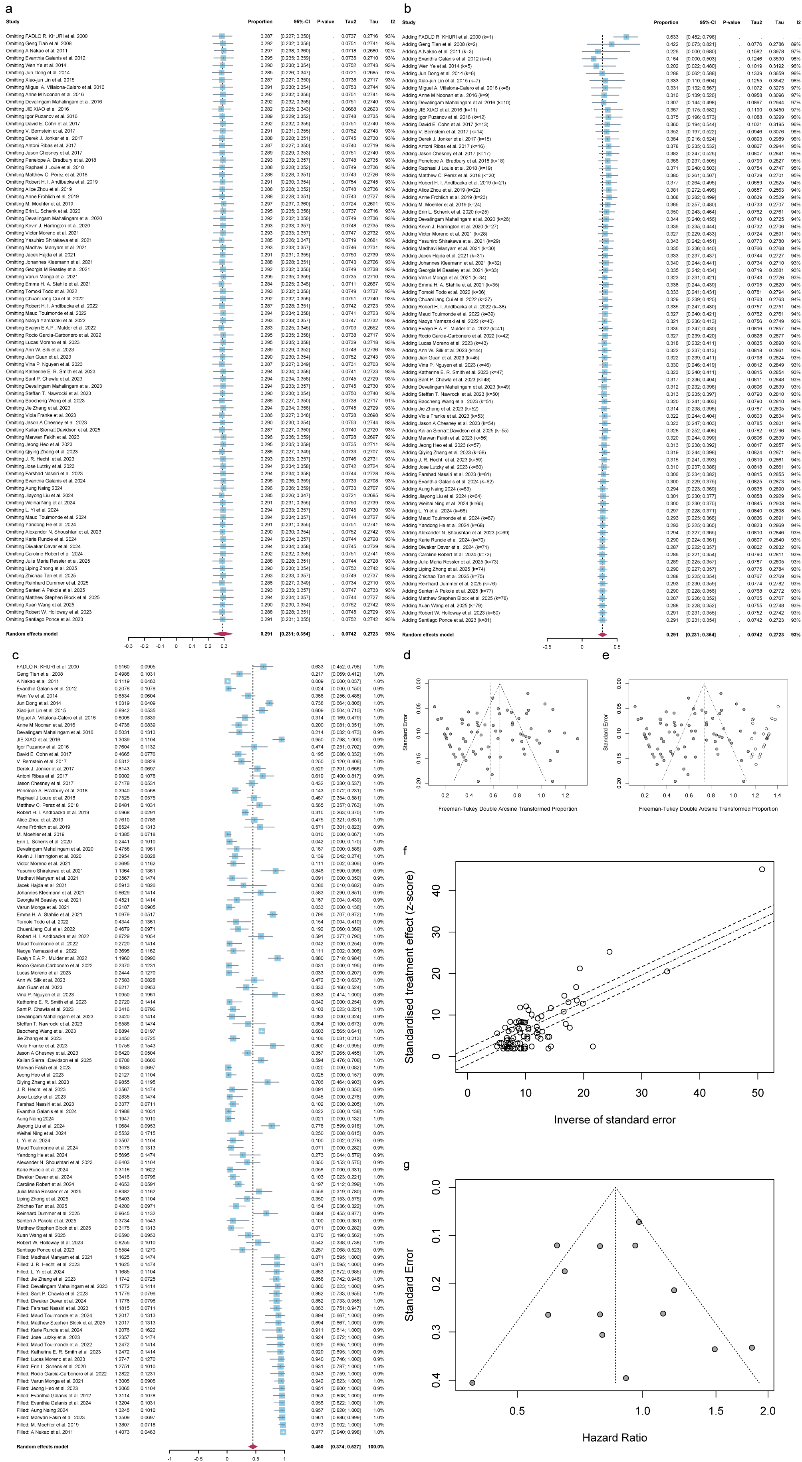


Figure S4: (a) Forest plot of sensitivity analysis of ORR. (b) Forest plot of cumulative meta-analysis of ORR. (c) Forest plot after trim and fill method of ORR. (d-e) Funnel plot after trim and fill method of ORR. (f) Galbraith plot of ORR. (g)Funnel plot of DRR.


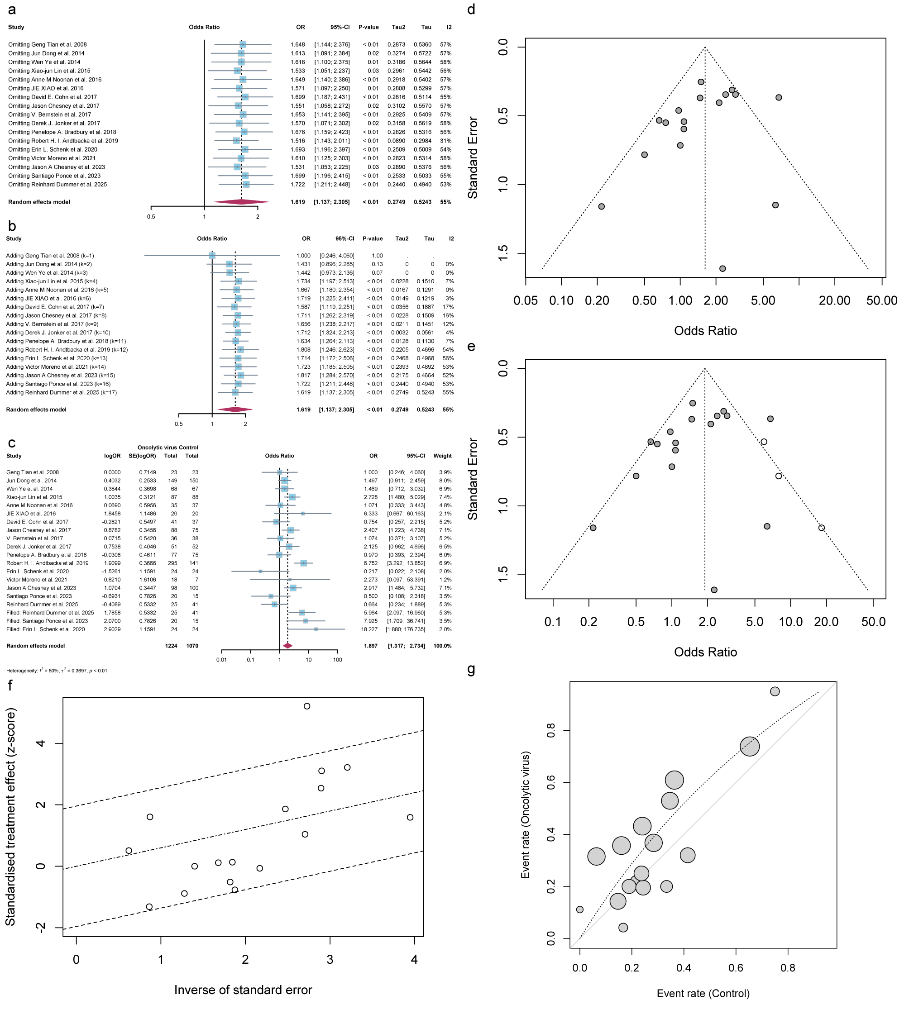


Figure S5: (a) Forest plot of sensitivity analysis of OR of ORR. (b) Forest plot of cumulative meta-analysis of OR of ORR. (c) Forest plot after trim and fill method of OR of ORR. (d-e) Funnel plot after trim and fill method of OR of ORR. (f) Galbraith plot of OR of ORR. (g) L'Abbé plot of OR of ORR.


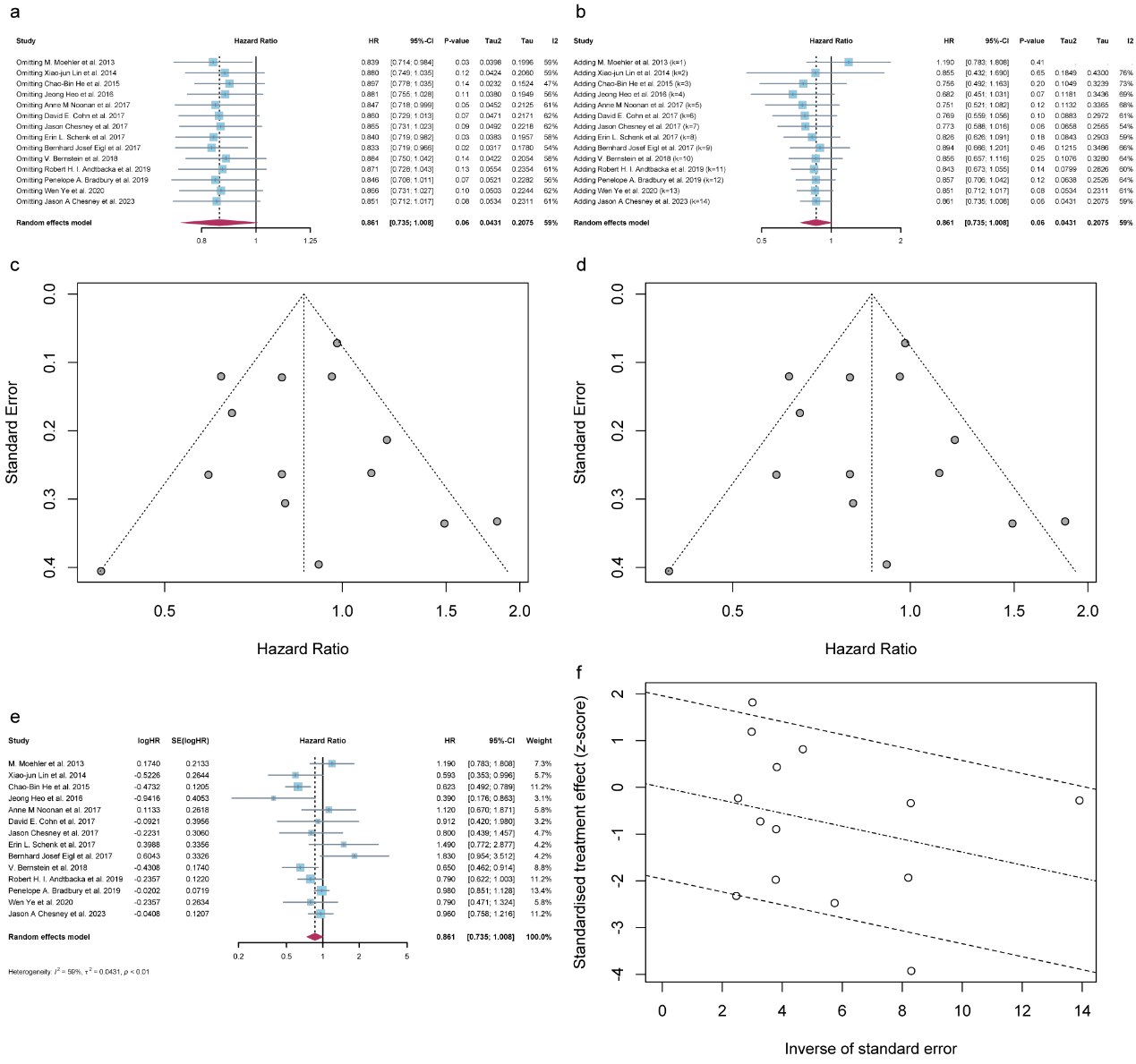


Figure S6: (a) Forest plot of sensitivity analysis of OS. (b) Forest plot of cumulative meta-analysis of OS. (c-d) Funnel plot after trim and fill method of OS. (e) Forest plot after trim and fill method of OS. (f) Galbraith plot of OS.


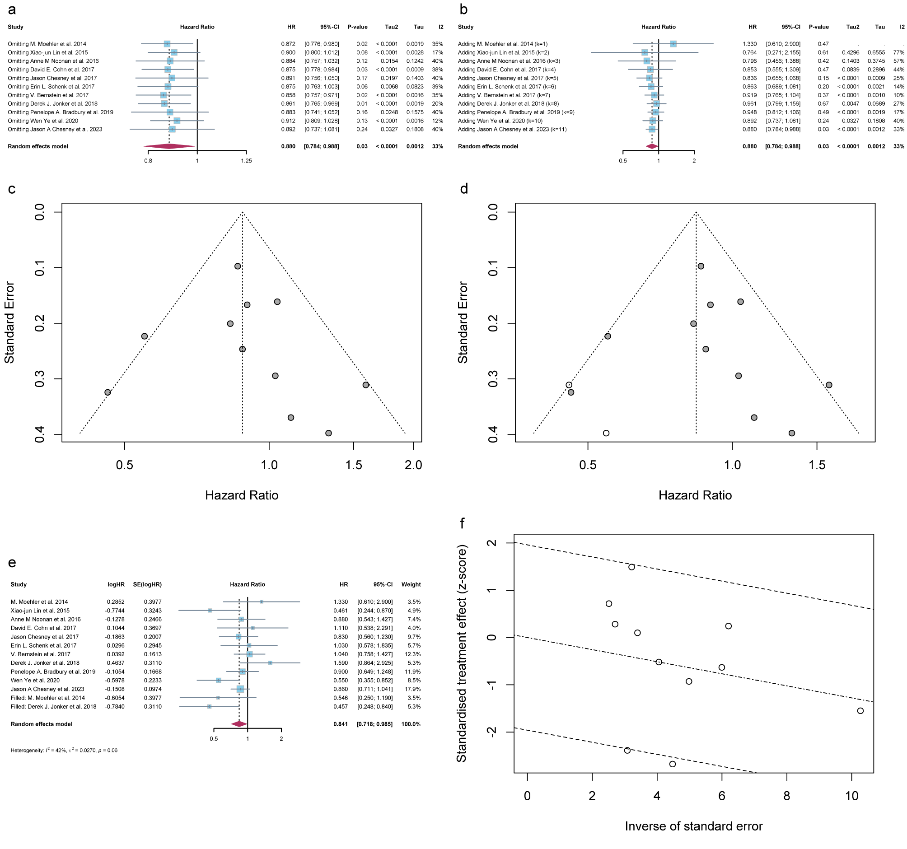


Figure S7: (a) Forest plot of sensitivity analysis of PFS. (b) Forest plot of cumulative meta-analysis of PFS. (c-d) Funnel plot after trim and fill method of PFS. (e) Forest plot after trim and fill method of PFS. (f) Galbraith plot of PFS.
