# Supplementary material for: Efficacy of oncolytic virus in the treatment of intermediate-to-advanced solid tumors: a systematic review and meta-analysis
Source: J Virol. 2025 Jun 20;99(7):e00640-25. doi: 10.1128/jvi.00640-25 (PMC12282134; doi:10.1128/jvi.00640-25)
Supplement: Table S1 — Details of search strategy. [file jvi.00640-25-s0002.docx]

**Details of Search Strategy**

**Pubmed**

| # | Search Terms | Hits |
| --- | --- | --- |
| #1 | Oncolytic Viruses[MeSH Terms] | 3240 |
| #2 | (((Oncolytic Viruses[MeSH Terms]) OR (Oncolytic Viruses[Title/Abstract])) OR (Oncolytic Virus[Title/Abstract])) OR (Oncolytic Virus[Title/Abstract])) OR (Viruses, Oncolytic[Title/Abstract]) | 5833 |
| #3 | Oncolytic Virotherapy[MeSH Terms] | 4286 |
| #4 | ((((((((((Oncolytic Virotherapy[MeSH Terms]) OR (Oncolytic Virotherapy[Title/Abstract])) OR (Oncolytic Virotherapies[Title/Abstract])) OR (Oncolytic Virotherapies[Title/Abstract])) OR (Virotherapy, Oncolytic[Title/Abstract])) OR (Oncolytic Virus Therapy[Title/Abstract])) OR (Oncolytic Virus Therapies[Title/Abstract])) OR (Therapies, Oncolytic Virus[Title/Abstract])) OR (Therapy, Oncolytic Virus[Title/Abstract])) OR (Virus Therapies, Oncolytic[Title/Abstract])) OR (Virus Therapy, Oncolytic[Title/Abstract]) | 5904 |
| #5 | Neoplasms[MeSH Terms] | 4081025 |
| #6 | ((((((((((((((((((Neoplasms[MeSH Terms]) OR (Neoplasms[Title/Abstract])) OR (Tumor[Title/Abstract])) OR (Neoplasm[Title/Abstract])) OR (Tumors[Title/Abstract])) OR (Neoplasia[Title/Abstract])) OR (Neoplasias[Title/Abstract])) OR (Cancer[Title/Abstract])) OR (Cancers[Title/Abstract])) OR (Malignant Neoplasm[Title/Abstract])) OR (Malignancy[Title/Abstract])) OR (Malignancies[Title/Abstract])) OR (Malignant Neoplasms[Title/Abstract])) OR (Neoplasm, Malignant[Title/Abstract])) OR (Neoplasms, Malignant[Title/Abstract])) OR (Benign Neoplasms[Title/Abstract])) OR (Benign Neoplasm[Title/Abstract])) OR (Neoplasms, Benign[Title/Abstract])) OR (Neoplasm, Benign[Title/Abstract]) | 5242790 |
| #7 | (#2 OR #4) AND #6 | 7092 |

**Cochrane**

| # | Search Terms | Hits |
| --- | --- | --- |
| #1 | MeSH descriptor: [Oncolytic Viruses] explode all trees | 28 |
| #2 | (Oncolytic Virus):ti,ab,kw or (Virus, Oncolytic):ti,ab,kw or (Viruses, Oncolytic):ti,ab,kw or (Oncolytic Viruses):ti,ab,kw | 136 |
| #3 | #1 or #2 | 136 |
| #4 | MeSH descriptor: [Oncolytic Virotherapy] explode all trees | 39 |
| #5 | (Oncolytic Virotherapy):ti,ab,kw or (Oncolytic Virotherapies):ti,ab,kw or (Virotherapies, Oncolytic):ti,ab,kw or (Virotherapy, Oncolytic):ti,ab,kw or (Oncolytic Virus Therapy):ti,ab,kw or (Oncolytic Virus Therapies):ti,ab,kw or (Therapies, Oncolytic Virus):ti,ab,kw or (Therapy, Oncolytic Virus):ti,ab,kw or (Virus Therapies, Oncolytic):ti,ab,kw or (Virus Therapy, Oncolytic):ti,ab,kw | 128 |
| #6 | #4 or #5 | 128 |
| #7 | MeSH descriptor: [Neoplasms] explode all trees | 126239 |
| #8 | (Neoplasms):ti,ab,kw or (Tumor):ti,ab,kw or (Neoplasm):ti,ab,kw or (Tumors):ti,ab,kw or (Neoplasia):ti,ab,kw or (Neoplasias):ti,ab,kw or (Cancer):ti,ab,kw or (Cancers):ti,ab,kw or (Malignant Neoplasm):ti,ab,kw or (Malignancy):ti,ab,kw or (Malignancies):ti,ab,kw or (Malignant Neoplasms):ti,ab,kw or (Neoplasm, Malignant):ti,ab,kw or (Neoplasms, Malignant):ti,ab,kw or (Benign Neoplasms):ti,ab,kw or (Benign Neoplasm):ti,ab,kw or (Neoplasms, Benign):ti,ab,kw or (Neoplasm, Benign):ti,ab,kw | 275832 |
| #9 | #7 or #8 | 289480 |
| #10 | (#3 or #6) and #9 | 150 |

**Embase**

| # | Search Terms | Hits |
| --- | --- | --- |
| #1 | oncolytic virus.mp. or exp oncolytic virus/ | 13531 |
| #2 | (oncolytic virus or oncolytic viruses or virus,oncolytic or oncolytic adenovirus or oncolytic herpes virus or oncolytic paramyxovirus or oncolytic parvovirus or oncolytic reovirus).ab,kw,ti | 8248 |
| #3 | oncolytic virus therapy.mp. or exp oncolytic virotherapy/ | 6019 |
| #4 | (oncolytic virus therapy or oncolytic viral therapy).ab,kw,ti | 801 |
| #5 | malignant neoplasm.mp. or exp malignant neoplasm/ | 4798301 |
| #6 | (malignant neoplasm or cancer or cancers or malignant neoplasia or malignant neoplastic disease or malignant tumor or malignant tumour or neoplasia, malignant or tumor, malignant or tumour, malignant or neoplasm or advanced cancer or bilateral cancer or childhood cancer or congenital cancer or disseminated cancer or drug induced cancer or early cancer or inoperable cancer or malignant neoplasms subdivided by anatomical site or mesothelioma or minimal residual disease or mucosal cancer or multiple cancer or occult cancer or occupational cancer or primary tumor or radiation induced cancer or second cancer or solid malignant neoplasm or superficial cancer or transmissible cancer).ab,kw,ti | 3632500 |
| #7 | #1 or #2 | 14333 |
| #8 | #3 or #4 | 6132 |
| #9 | (#7 or #8) and (#5 or #6) | 14051 |

**Medline**

| # | Search Terms | Hits |
| --- | --- | --- |
| #1 | oncolytic virus.mp. or exp oncolytic virus/ | 4686 |
| #2 | (oncolytic virus or oncolytic viruses or virus,oncolytic or oncolytic adenovirus or oncolytic herpes virus or oncolytic paramyxovirus or oncolytic parvovirus or oncolytic reovirus).ab,kw,ti | 5609 |
| #3 | oncolytic virus therapy.mp. or exp oncolytic virotherapy/ | 4418 |
| #4 | (oncolytic virus therapy or oncolytic viral therapy).ab,kw,ti | 571 |
| #5 | malignant neoplasm.mp. or exp malignant neoplasm/ | 4083012 |
| #6 | (malignant neoplasm or cancer or cancers or malignant neoplasia or malignant neoplastic disease or malignant tumor or malignant tumour or neoplasia, malignant or tumor, malignant or tumour, malignant or neoplasm or advanced cancer or bilateral cancer or childhood cancer or congenital cancer or disseminated cancer or drug induced cancer or early cancer or inoperable cancer or malignant neoplasms subdivided by anatomical site or mesothelioma or minimal residual disease or mucosal cancer or multiple cancer or occult cancer or occupational cancer or primary tumor or radiation induced cancer or second cancer or solid malignant neoplasm or superficial cancer or transmissible cancer).ab,kw,ti | 2571743 |
| #7 | #1 or #2 | 6465 |
| #8 | #3 or #4 | 4561 |
| #9 | (#7 or #8) and (#5 or #6) | 6861 |
