# Supplementary material for: Efficacy of oncolytic virus in the treatment of intermediate-to-advanced solid tumors: a systematic review and meta-analysis
Source: J Virol. 2025 Jun 20;99(7):e00640-25. doi: 10.1128/jvi.00640-25 (PMC12282134; doi:10.1128/jvi.00640-25)
Supplement: Table S2 — Inclusion/exclusion criteria. [file jvi.00640-25-s0003.docx]

**Supplementary Table 2. the Inclusion/Exclusion Criteria**

| **Inclusion Criteria (PICOS)** | |
| --- | --- |
| **Category** | **Content** |
| **Patients** | Individuals diagnosed with intermediate and advanced solid tumors, irrespective of nationality, gender, or race. |
| **Interventions** | Patients who received single-agent therapy or combination therapy with oncolytic viruses (OVs). |
| **Comparators** | Patients who received treatments other than OVs therapy. |
| **Outcomes** | Studies should report at least one of the following outcomes: Complete response (CR), partial response (PR), stable disease (SD), progressive disease (PD); Overall response rate (ORR), durable response rate (DRR), overall survival (OS), and progression-free survival (PFS). |
| **Study Design** | Retrospective studies or clinical trials. |
| **Exclusion Criteria** | |
| Unpublished studies. | |
| Duplicated data. | |
| Patients who did not receive OVs treatment for the first time. | |
