# Supplementary material for: Efficacy of oncolytic virus in the treatment of intermediate-to-advanced solid tumors: a systematic review and meta-analysis
Source: J Virol. 2025 Jun 20;99(7):e00640-25. doi: 10.1128/jvi.00640-25 (PMC12282134; doi:10.1128/jvi.00640-25)
Supplement: Table S3 — JADAD scale for assessing the quality of clinical trials. [file jvi.00640-25-s0004.docx]

Supplementary Table 3. The JADAD Scale for assessing the quality of clinical trials.

| **Study (Year)** | **Randomization** | **Concealment of allocation** | **Double blinding** | **Withdraws and dropouts** | **Overall score** |
| --- | --- | --- | --- | --- | --- |
| Anne M Noonan et al. (2016) | 2 | 2 | 0 | 1 | 5 |
| Bernhard Josef Eigl et al. (2017) | 2 | 1 | 0 | 2 | 5 |
| David E. Cohn et al. (2017) | 2 | 2 | 0 | 1 | 5 |
| Derek J. Jonker et al. （2017） | 2 | 1 | 0 | 2 | 5 |
| Erin L. Schenk et al. (2020) | 2 | 1 | 2 | 2 | 7 |
| Geng Tian et al. （2008） | 1 | 1 | 0 | 2 | 4 |
| Jason Chesney et al. (2017) | 2 | 2 | 0 | 1 | 5 |
| Jeong Heo et al. (2013) | 1 | 1 | 1 | 1 | 4 |
| JIE XIAO et al. (2016) | 2 | 1 | 0 | 2 | 5 |
| M. Moehler et al. (2019) | 1 | 1 | 1 | 1 | 4 |
| Maud Toulmonde et al. (2022) | 1 | 1 | 1 | 1 | 4 |
| Penelope A. Bradbury et al. (2018) | 2 | 1 | 0 | 2 | 5 |
| Robert H. I. Andtbacka et al. (2019) | 1 | 1 | 1 | 1 | 4 |
| V. Bernstein et al. (2017) | 2 | 1 | 1 | 2 | 6 |
| Victor Moreno et al. (2021) | 1 | 1 | 1 | 1 | 4 |
| Wen Ye et al. (2014) | 2 | 1 | 0 | 2 | 5 |
| Reinhard Dummer et al. (2023) | 2 | 2 | 1 | 1 | 6 |
| Jason A Chesney et al. (2023) | 2 | 2 | 0 | 1 | 5 |
| Santiago Ponce et al. (2023) | 1 | 1 | 1 | 1 | 4 |
| Reinhard Dummer et al. (2025) | 1 | 1 | 1 | 1 | 4 |
