# Supplementary material for: Efficacy of oncolytic virus in the treatment of intermediate-to-advanced solid tumors: a systematic review and meta-analysis
Source: J Virol. 2025 Jun 20;99(7):e00640-25. doi: 10.1128/jvi.00640-25 (PMC12282134; doi:10.1128/jvi.00640-25)
Supplement: Table S4 — Minors scale for assessing the quality of single-arm clinical trials. [file jvi.00640-25-s0005.docx]

**Supplementary Table 4. The Minors Scale for assessing the quality of single arm clinical trials.**

| **Study (Year)** | **A clearly stated aim** | **Inclusion of consecutive patients** | **Prospective collection of data** | **Endpoints appropriate to the aim of the study** | **Unbiased assessment of the study endpoint** | **Follow-up period appropriate to the aim of the study** | **Loss to follow up less than 5%** | **Prospective calculation of the study size** | **Overall Score** |
| --- | --- | --- | --- | --- | --- | --- | --- | --- | --- |
| A Nakao et al. (2011) | 2 | 2 | 1 | 1 | 1 | 1 | 2 | 0 | 10 |
| Antoni Ribas et al. (2017) | 2 | 2 | 2 | 2 | 2 | 0 | 2 | 0 | 12 |
| ChuanLiang Cui et al. (2022) | 2 | 2 | 1 | 1 | 1 | 1 | 2 | 0 | 10 |
| Devalingam Mahalingam et al. (2016) | 2 | 2 | 2 | 1 | 2 | 1 | 2 | 0 | 12 |
| Devalingam Mahalingam et al. (2020) | 2 | 2 | 2 | 2 | 2 | 2 | 0 | 0 | 12 |
| Evanthia Galanis et al. (2012) | 2 |  | 2 | 1 | 2 | 2 | 2 | 0 | 11 |
| FADLO R. KHURI et al. (2000) | 2 | 2 | 2 | 1 | 1 | 1 | 2 | 0 | 11 |
| Georgia M Beasley et al. (2021) | 2 | 2 | 1 | 1 | 1 | 1 | 1 | 0 | 9 |
| Hatem Soliman et al. (2020) | 2 | 2 | 1 | 1 | 1 | 1 | 2 | 0 | 10 |
| Igor Puzanov et al. (2016) | 2 | 2 | 2 | 1 | 1 | 1 | 2 | 0 | 11 |
| Jacek Hajda et al. (2021) | 2 | 2 | 1 | 1 | 1 | 1 | 2 | 0 | 10 |
| Kevin J. Harrington et al. (2020) | 2 | 2 | 2 | 1 | 1 | 1 | 2 | 0 | 11 |
| Madhavi Manyam et al. (2021) | 2 | 2 | 1 | 1 | 1 | 1 | 2 | 0 | 10 |
| Miguel A. Villalona-Calero et al. (2016) | 2 | 2 | 2 | 2 | 2 | 2 | 0 | 0 | 12 |
| Naoya Yamazaki et al. (2022) | 2 | 2 | 1 | 1 | 1 | 1 | 2 | 0 | 10 |
| Robert H. I. Andtbacka et al. (2022) | 2 | 2 | 1 | 1 | 1 | 1 | 2 | 0 | 10 |
| Rocio Garcia-Carbonero et al. (2022) | 2 | 2 | 1 | 1 | 1 | 1 | 1 | 0 | 9 |
| Tomoki Todo et al. (2022) | 2 | 2 | 1 | 1 | 1 | 1 | 1 | 0 | 9 |
| Varun Monga et al. (2021) | 2 | 2 | 1 | 1 | 1 | 1 | 2 | 0 | 10 |
| Yasuhiro Shirakawa et al. (2021) | 2 | 2 | 1 | 1 | 1 | 1 | 2 | 0 | 10 |
| Lucas Moreno et al. (2023) | 2 | 2 | 1 | 1 | 1 | 1 | 2 | 0 | 10 |
| Ann W. Silk et al. (2023) | 2 | 2 | 2 | 1 | 1 | 1 | 2 | 0 | 11 |
| Jian Guan et al. (2023) | 2 | 2 | 1 | 1 | 1 | 1 | 2 | 0 | 10 |
| Vina P. Nguyen et al.(2023) | 2 | 2 | 1 | 1 | 1 | 1 | 2 | 0 | 10 |
| Katherine E. R. Smith et al. (2023) | 2 | 2 | 1 | 1 | 1 | 1 | 1 | 0 | 9 |
| Sant P. Chawla et al. (2023) | 2 | 2 | 1 | 1 | 1 | 1 | 2 | 0 | 10 |
| Robert W. Holloway et al. (2023) | 2 | 2 | 1 | 1 | 1 | 1 | 2 | 0 | 10 |
| Alexander L. Ling et al. (2023) | 2 | 2 | 1 | 1 | 1 | 1 | 2 | 0 | 10 |
| Devalingam Mahalingam et al. (2023) | 2 | 2 | 1 | 1 | 1 | 1 | 1 | 0 | 9 |
| Steffan T. Nawrocki et al. (2023) | 2 | 2 | 2 | 1 | 1 | 1 | 2 | 0 | 11 |
| Jie Zhang et al. (2023) | 2 | 2 | 1 | 1 | 1 | 1 | 2 | 0 | 10 |
| Viola Franke et al. (2023) | 2 | 2 | 1 | 1 | 1 | 1 | 2 | 0 | 10 |
| Marwan Fakih et al. (2023) | 2 | 2 | 2 | 1 | 1 | 1 | 2 | 0 | 11 |
| Jeong Heo et al. (2023) | 2 | 2 | 1 | 1 | 1 | 1 | 1 | 0 | 9 |
| Qiying Zhang et al. (2023) | 2 | 2 | 1 | 1 | 1 | 1 | 2 | 0 | 10 |
| J. R. Hecht et al. (2023) | 2 | 2 | 2 | 1 | 1 | 1 | 2 | 0 | 11 |
| Jose Lutzky et al. (2023) | 2 | 2 | 1 | 1 | 1 | 1 | 2 | 0 | 10 |
| Farshad Nassiri et al. (2023) | 2 | 2 | 1 | 1 | 1 | 1 | 2 | 0 | 10 |
| Alexander N. Shoushtari et al. (2023) | 2 | 2 | 1 | 1 | 1 | 1 | 2 | 0 | 10 |
| Evanthia Galanis et al. (2024) | 2 | 2 | 1 | 1 | 1 | 1 | 1 | 0 | 9 |
| Aung Naing et al. (2024) | 2 | 2 | 2 | 1 | 1 | 1 | 2 | 0 | 11 |
| Jiayong Liu et al. (2024) | 2 | 2 | 1 | 1 | 1 | 1 | 2 | 0 | 10 |
| Weihai Ning et al. (2024) | 2 | 2 | 1 | 1 | 1 | 1 | 2 | 0 | 10 |
| L. Yi et al. (2024) | 2 | 2 | 2 | 1 | 1 | 1 | 2 | 0 | 11 |
| Maud Toulmonde et al. (2024) | 2 | 2 | 1 | 1 | 1 | 1 | 1 | 0 | 9 |
| Yandong He et al. (2024) | 2 | 2 | 1 | 1 | 1 | 1 | 2 | 0 | 10 |
| Karie Runcie et al. (2024) | 2 | 2 | 1 | 1 | 1 | 1 | 1 | 0 | 9 |
| Diwakar Davar et al. (2024) | 2 | 2 | 2 | 1 | 1 | 1 | 2 | 0 | 11 |
| Caroline Robert et al. (2024) | 2 | 2 | 1 | 1 | 1 | 1 | 2 | 0 | 10 |
| Julia Maria Ressler et al. (2025) | 2 | 2 | 1 | 1 | 1 | 1 | 2 | 0 | 10 |
| Liping Zhong et al. (2025) | 2 | 2 | 2 | 1 | 1 | 1 | 2 | 0 | 11 |
| Zhichao Tan et al. (2025) | 2 | 2 | 2 | 1 | 1 | 1 | 2 | 0 | 11 |
| Santeri A Pakola et al. (2025) | 2 | 2 | 1 | 1 | 1 | 1 | 2 | 0 | 10 |
| Matthew Stephen Block et al. (2025) | 2 | 2 | 1 | 1 | 1 | 1 | 2 | 0 | 10 |
| Xuan Wang et al. (2025) | 2 | 2 | 2 | 1 | 1 | 1 | 2 | 0 | 11 |
