# Supplementary material for: Efficacy of oncolytic virus in the treatment of intermediate-to-advanced solid tumors: a systematic review and meta-analysis
Source: J Virol. 2025 Jun 20;99(7):e00640-25. doi: 10.1128/jvi.00640-25 (PMC12282134; doi:10.1128/jvi.00640-25)
Supplement: Table S5 — Newcastle-Ottawa scale (NOS) for assessing the quality of cohort studies. [file jvi.00640-25-s0006.doc]

**Supplementary Table 5**. The Newcastle-Ottawa Scale (NOS) for assessing the quality of cohort studies.

| Study | selection | | | | Comparability | Outcome | | | score |
| --- | --- | --- | --- | --- | --- | --- | --- | --- | --- |
|  | Representativeness of the exposed cohort | Selection of the non exposed cohort | Ascertainment of exposure | Demonstration that outcome of interest was not present at start of study | Comparability of cohorts on the basis of the design or analysis | Assessment of outcome | Was follow-up long enough for outcomes to occur | Adequacy of follow up of cohorts |  |
| Raphael J Louie et al. (2018) | * | / | * | * | * | * | * | * | 7 |
| Matthew C. Perez et al. (2018) | * | / | * | * | * | * | * | * | 7 |
| Alice Zhou et al. (2019) | * | * | * | * | * | * | * | * | 8 |
| Anne Fröhlich et al. (2019) | * | * | * | * | * | * | * | * | 8 |
| Jun Dong et al. (2014) | * | * | * | / | * | * | * | ** | 8 |
| Xiao-jun Lin et al. (2015) | * | * | * | / | * | * | * | ** | 8 |
| Ying-wei Zhu et al. (2014) | * | * | * | / | * | * | * | ** | 8 |
| Chao-Bin He et al. (2017) | * | * | * | / | * | * | * | * | 7 |
| Ran Zhang et al. (2019) | * | * | * | / | * | * | * | * | 7 |
| Emma H. A. Stahlie et al. (2021) | * | * | * | / | * | * | * | * | 7 |
| Johannes Kleemann et al. (2021) | * | * | * | / | * | * | * | * | 7 |
| Evalyn E.A.P . Mulder et al. (2022) | * | * | * | / | * | * | * | ** | 8 |
| Baocheng Wang et al. (2023) | * | * | * | / | * | * | * | * | 7 |
| Kailan Sierra‑Davidson et al. (2025) | * | * | * | / | * | * | * | * | 7 |
