# Supplementary material for: Efficacy of oncolytic virus in the treatment of intermediate-to-advanced solid tumors: a systematic review and meta-analysis
Source: J Virol. 2025 Jun 20;99(7):e00640-25. doi: 10.1128/jvi.00640-25 (PMC12282134; doi:10.1128/jvi.00640-25)
Supplement: Table S6 — Meta-regression analysis of factors affecting heterogeneity for the association between OVs use and cancer risk. [file jvi.00640-25-s0007.docx]

**Table S6. Meta-regression analysis of factors affecting heterogeneity for the association between OVs use and cancer risk.**

1. **ORR**

| **Variable** | **Coefficient (95%CI)** | **SE** | **Statistical significance (p)** |
| --- | --- | --- | --- |
| **Publication of year** | -0.0088 (-0.0235, 0.0059) | 0.0075 | 0.2385 |
| **Age** | 0.0065 (-0.0008, 0.0137) | 0.0037 | 0.0811 |
| **The proportion of male** | -0.0016 (-0.0048, 0.0017) | 0.0016 | 0.3429 |

1. **OR of ORR**

| **Variable** | **Coefficient (95%CI)** | **SE** | **Statistical significance (p)** |
| --- | --- | --- | --- |
| **Publication of year** | -0.0132 (-0.1112, 0.0849) | 0.0500 | 0.7920 |
| **Age** | -0.0363 (-0.1138, 0.0412) | 0.0395 | 0.3587 |
| **The proportion of male** | 0.0057 (-0.0094, 0.0208) | 0.0077 | 0.4570 |

**OVs, oncolytic viruses; CI, Confidence Interval; se, SE, standard error**
